# Palliative extubation in intensive care units: scoping review

**DOI:** 10.1590/1980-220X-REEUSP-2025-0409en

**Published:** 2026-03-16

**Authors:** Rafaella Guilherme Gonçalves, Lucas Batista Ferreira, Tarcísio Tércio das Neves, Carlos Jordão de Assis Silva, Fabiane Rocha Botarelli, Rejane Maria Paiva de Menezes

**Affiliations:** 1 Universidade Federal do Rio Grande do Norte, Departamento de Enfermagem, Natal, RN, Brazil.

**Keywords:** Palliative Care, Intensive Care Units, Patient Care Team, Airway Extubation, Nursing

## Abstract

**Objective:**

To map existing knowledge on palliative extubation in adult patients admitted to intensive care units.

**Method:**

Scoping review conducted according to JBI guidelines. Searches were conducted between October and December 2024 in seven databases and four repositories, in addition to a reverse search. The selection was independent, the extraction followed a structured protocol, and the report used PRISMA-ScR. The analysis of the results was based on the PAGER framework (*Patterns, Advances, Gaps, Evidence for practice and Research recommendations*).

**Results:**

The search identified 1,463 materials and included 44 studies. Publications in 2023 predominated. The findings addressed recommendations for practice, time to death, comparison between terminal extubation and terminal weaning, symptom management, experiences and challenges of professionals, as well as aspects related to decision-making and the use of protocols in palliative extubation.

**Conclusion:**

Palliative extubation remains marked by heterogeneous practices and limited production of high-level evidence. There is a need for evidence-based protocols and multidisciplinary training to ensure quality care, dignity, and relief from suffering at the end of life.

## INTRODUCTION

Patients with life-threatening diseases or conditions may progress to critical conditions characterized by instability or imminent risk of organ failure and death. In these situations, care usually takes place in Intensive Care Units (ICUs) or Intermediate Care Units (ICUs), environments prepared to offer highly complex life support, with continuous monitoring and resources aimed at maintaining life^(1)^. Despite technological advances, mortality in Brazilian ICUs remains high, ranging from 9.6% to 58%, influenced by factors such as clinical severity, comorbidities, length of stay, mechanical ventilation, sepsis, acute renal failure, and quality of care^(2)^.

The early integration of palliative care (PC) into disease-modifying treatment is essential for the comprehensive care of critically ill patients, helping to avoid futile interventions, reduce length of stay and costs, alleviate suffering, and preserve dignity, without hastening the dying process^(3)^. When incorporated concomitantly, PC favors ethical and humanized decisions, with a focus on strict symptom control^(2)^.

In addition, the early introduction of PC favors the definition of Life Support Limits (LSL), allowing professionals and family members to participate in decisions about the therapeutic plan and monitor the progression of PC until the end-of-life phase^(4)^. LSL consists of not initiating, suspending, or limiting life-support interventions in irreversible situations, avoiding futile treatments that artificially prolong the dying process^(5)^. In the ICU setting, the discussion is particularly relevant, including decisions about hemodialysis, vasoactive drugs, artificial nutrition, and invasive mechanical ventilation, the latter being generally the final measure to be discontinued, as its withdrawal usually determines death in the short term^(6,7)^.

Palliative extubation (PE) refers to the planned withdrawal of mechanical ventilation in patients with imminent death and is recognized as an ethical and morally justifiable practice because it avoids dysthanasia and allows death with comfort and dignity^(8)^. This procedure requires technical preparation, effective communication, and qualified multidisciplinary action to ensure symptom control and adequate support for the patient and family^(9)^.

However, the implementation of PE still faces significant challenges. Among them are cultural, religious, and emotional barriers, the absence of clear institutional protocols, and the heterogeneity of conduct among teams^(10)^.

In the Brazilian context, the difficulties are aggravated by gaps in academic and professional training on the process of dying and death, insufficient training in communicating bad news, and structural limitations for integrating PE into intensive care^(5)^. Thus, PE is one of the most complex and sensitive LSL behaviors, requiring technical, ethical, and emotional preparation of health teams.

Given this scenario, it is essential to broaden the debate and reflections on the role of health professionals in the proper management of PE, with special attention to nursing, which plays a central role in promoting the physical, psychological, social, and spiritual comfort of patients at the end of life. Thus, this study aims to map existing knowledge about palliative extubation in adult patients admitted to intensive care units, synthesizing the findings according to the PAGER (Patterns, Advances, Gaps, Evidence for practice and Research recommendations) framework.

## METHOD

### Study Design

This is a scoping review that sought to explore, map, synthesize scientific evidence, and identify gaps in knowledge about PE in adult patients admitted to the ICU. The method followed the JBI guidelines for scoping reviews^(11)^ and the writing complied with the PRISMA-ScR recommendations^(12)^. The protocol for this review was registered in the Open Science Framework (DOI: 10.17605/OSF.IO/JYMNZ).

### Data Collection

Initially, a preliminary search was conducted in the MEDLINE and Scopus databases to identify studies related to the topic and select keywords and index terms used in these publications. To develop the guiding question, the acronym PCC, described in Chart 1, was adopted.

**Chart 1:** PCC Strategy (Population, Concept, and Context) – Natal, RN, Brazil, 2025.

Acronym	Term	Description
P	Population	Adult patients in end-of-life care
C	Concept	Palliative extubation
C	Context	Intensive Care Unit

Based on this strategy, the following research question was formulated: “What knowledge exists in the literature on palliative extubation of adult patients admitted to intensive care units?”

The bibliographic survey was conducted between October and December 2024 through the journal portal of the Coordination for the Improvement of Higher Education Personnel (CAPES), via the Federated Academic Community (CAFe), covering the SCOPUS (Elsevier), MEDLINE/PubMed (National Library of Medicine), *Web of Science*, CINAHL (Cumulative Index to Nursing and Allied Health Literature), Google Scholar, LILACS (Latin American and Caribbean Health Sciences Literature), and BDENF (Nursing Database). In addition, a reverse search was performed on the references of the included articles to identify additional relevant studies. To broaden the scope, gray literature sources were also consulted, including theses and dissertations available in online repositories: CAPES Thesis and Dissertation Catalog, BASE (Bielefeld Academic Search Engine), Cybertesis, and Open Access Theses & Dissertations (OATD).

The descriptors selected for the search were the terms from the Health Sciences Descriptors (DeCS) and the corresponding terms in the Medical Subject Headings (MESH): Palliative Care, Airway Extubation, and Intensive Care Units, in addition to the keywords: Terminal Care, End-of-life care, Compassionate Extubation, Terminal weaning, and Terminal extubation, and the keyword: Palliative Extubation.

The search strategies were developed and adapted according to the syntactic and operational specificities of each database, respecting the respective indexing fields, the use of Boolean operators, and the specific information retrieval resources. Chart 2 presents the search strategies adopted in each database.

**Chart 2:** Search strategy used for each database – Natal, RN, Brazil, 2025.

Database	Strategy
SCOPUS	(“Palliative Care” OR “Terminal Care” OR “End-of-life Care”) AND (“Airway Extubation” OR “Compassionate Extubation” OR “Terminal Extubation” OR “Terminal Weaning”) AND (“Intensive Care Units”)
MEDLINE/PubMed	(Palliative Care OR Terminal Care OR End-of-life Care) AND (Airway Extubation OR Compassionate Extubation OR Terminal Extubation OR Terminal Weaning) AND (Intensive Care Units)
Web of Science	ALL=(Palliative Care OR Terminal Care OR End-of-life Care) AND ALL=(Airway Extubation OR Compassionate Extubation OR Terminal Extubation OR Terminal Weaning) AND ALL=(Intensive Care Units)
CINAHL	(Palliative Care OR Terminal Care OR End-of-life Care) AND (Airway Extubation OR Compassionate Extubation OR Terminal Extubation OR TerminalWeaning) AND (Intensive Care Units)
Google Scholar	(“Palliative Care” OR “Terminal Care” OR “End-of-life Care”) AND (“Airway Extubation” OR “Compassionate Extubation” OR “Terminal Extubation” OR “Terminal Weaning”) AND (“Intensive Care Units”)
LILACS	Palliative Care AND Airway Extubation
BDENF	Palliative Care AND Airway Extubation
**Gray literature**	**Strategy**
Catálogo de teses e dissertações da CAPES	Extubação Paliativa
BASE	Palliative Care AND Airway Extubation
Cybertesis	Palliative Care AND Airway Extubation
OATD	Palliative Care AND Airway Extubation AND Intensive Care Units

### Selection Criteria

The selection criteria were established based on the guiding question, considering the PCC strategy. Studies with the following characteristics were considered: a) regarding the population – studies conducted with patients in end-of-life care, aged 18 years or older; b) regarding the concept – studies that evaluated or described the performance of PE; c) regarding the context – studies that contemplate PE performed in ICUs. In addition, primary research studies with quantitative, qualitative, and mixed-method designs, review studies, and clinical opinion studies were included, available in full, without language restrictions and without time limits. Abstracts and letters to the editor were excluded.

### Data Extraction And Analysis

The selection of studies was conducted by two reviewers, independently and blinded, in two phases: screening of titles/abstracts and reading of potentially eligible texts in full.

Duplicates were removed and, in case of disagreement, a third reviewer was consulted. Additionally, a reverse search was performed on the references of the included studies. The identification, screening, and inclusion process is detailed in a flowchart according to the PRISMA-ScR guideline^(12)^.

For data extraction, a collection instrument was developed containing the variables of interest: authors, year of publication, country, objectives, method, population and sample size, outcomes, and main results related to the review question. The extracted information was organized in a spreadsheet and presented using descriptive statistics. In addition, a summary table was developed to facilitate the visualization and synthesis of the findings.

For the analysis, the PAGER (Patterns, Advances, Gaps, Evidence for practice and Research recommendations) methodology was used, which enhances the quality and rigor of scope reviews through a structured analysis of the findings. This approach includes: (1) Patterns – main emerging themes; (2) Advances – new discoveries, theoretical or methodological insights; (3) Gaps – areas that require further investigation; (4) Evidence for practice – findings relevant to practical application; and (5) Research recommendations – directions for future studies^(13)^.

### Ethical Aspects

The research was conducted in accordance with applicable ethical and methodological principles, with respect for good scientific practices and the copyright of the works consulted. As this review was based on publicly available secondary data, without direct involvement of human subjects, there was no need to submit it to a research ethics committee.

## RESULTS

**Figure 1:**
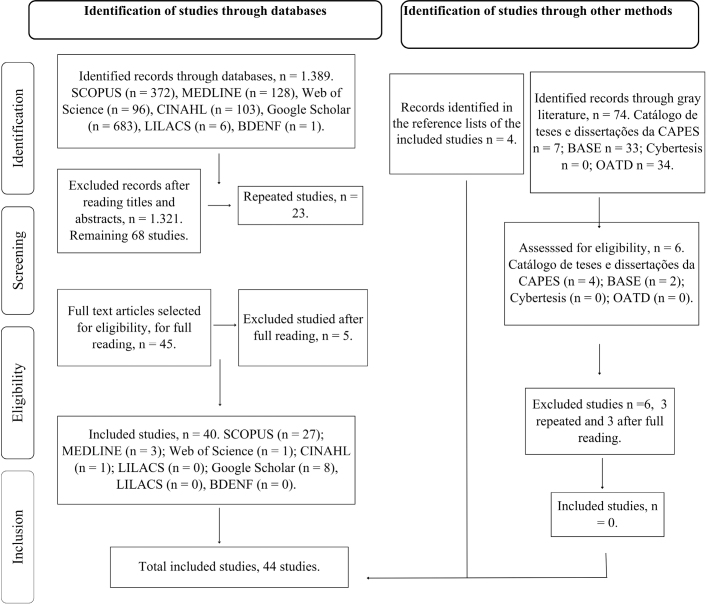
PRISMA-ScR flowchart for publication selection – Natal, RN, Brazil, 2025.

The search conducted between October and December 2024 identified 1,463 records, 1,389 from databases and 74 from gray literature. After screening titles and abstracts, 1,389 studies were excluded for not meeting the eligibility criteria, leaving 74 for full-text reading. In the next step, 26 duplicates were removed (23 from the databases and 3 from the gray literature), totaling 48 eligible studies. Of these, eight were excluded because they did not present the scenario of implementation or because they deviated from the proposed theme. After full reading, 40 studies were included, plus four identified by manual search in the references. Thus, the final sample comprised 44 studies, as illustrated in the PRISMA-ScR flowchart (Figure 1).

The 44 studies included in this scoping review consist of publications from the last 25 years, between 1999 and 2024, with a predominance of seven studies (15.9%) published in 2023. In addition, 2024 already looks promising for research on the topic, with six publications (13.6%) identified for that period. This information is presented in Chart 3.

**Chart 3:** Characterization of studies in terms of author, year, country of origin, objective, study design, population and main results – Natal, RN, Brazil, 2025.

Id	Authors/Year/Country	Type of study, population and sample	Main results
E1	Jennerich, 2024^(3)^. USA	Literature review, 83 studies	Adequate symptom control and team preparation are essential to minimize suffering during and after PE.
E2	Schaden et al., 2024^(14)^. Austria	Literature review, does not describe	Early transition to comfort care favors dignity at the end of life; PE requires rigorous preparation.
E3	Kumar et al., 2024^(15)^. India	Literature review, 15 studies	PE and terminal weaning are ethical options when aligned with the interests of the terminally ill patient.
E4	Curtisi et al., 2024^(16)^. USA	Case report, 2 patients	Gradual weaning associated with adequate symptom management provided comfort, while abrupt extubation with insufficient medication resulted in suffering.
E5	Marica, 2024^(17)^. USA	Intervention Study	A multidisciplinary, evidence-based guideline for PE increases professional safety and quality of care.
E6	Bryan et al., 2024^(18)^. USA	Prospective observational, with a qualitative approach, 312 health professionals	Communication and multi-professional integration, combined with the use of protocols, are fundamental for a safe and humanized PE, with the team-family relationship being central to the end-of-life decision-making process.
E7	Waldauf et al., 2023^(19)^. Canada, Czechia and the Netherlands	Prospective observational study, 616 patients	The decision for PE was influenced by the institutional context and associated with less ventilatory support and shorter survival.
E8	Zheng et al., 2023^(20)^. Taiwan	Retrospective observational study, 140 patients	The time to death after PE was variable, being shorter in patients with greater clinical severity, which helps in the planning of palliative care.
E9	Mazzu et al., 2023^(21)^. USA	Literature review, 73 studies	Structured communication and team support are key practices; robust evidence is still limited.
E10	Ortega-Chen et al., 2023^(10)^. USA	Literature review, does not describe	PE is a complex and variable process, requiring palliative communication skills, careful planning, symptom assessment and management, and debriefing.
E11	Roberts and Dahlin, 2023^(22)^. USA	Narrative review, does not describe	Nurses have a central role and can lead evidence-based practices in PE.
E12	Bastos et al., 2023^(23)^. Brazil	Descriptive, with a qualitative approach, health professionals	Professionals recognize the benefits of PE, but report technical insecurity and the need for training.
E13	Antonio and Antonio, 2023^(24)^. Brazil	Prospective observational study, 18 patients	Implementation of a local protocol organized practice and showed variability in time to death.
E14	Kaur et al., 2022^(25)^. USA	Cross-sectional, 20 health professionals	PE was associated with significant emotional distress among health professionals, especially among young women and those dissatisfied with the institutional process.
E15	Orr et al., 2022^(26)^ USA	Descriptive, with a qualitative approach, 20 health professionals - doctors, nurses and physiotherapists.	Professional experiences influence the conduct of PE; training and support reduce conflicts.
E16	Cooney-Newton and Hare, 2022^(27)^. USA	Literature review, does not describe	The Respiratory Distress Observation Scale is an objective tool that aids in the titration of comfort medications during end-of-life care and PE.
E17	Correa-Pérez, 2022^(28)^. Spain	Literature review, does not describe	Institutional protocols are needed for sedation, analgesia and family support.
E18	Dias, 2021^(29)^. Brazil	Integrative review, 20 studies	Nursing is fundamental in decision-making, performing PE and maintaining comfort.
E19	Lacerda et al., 2020^(30)^. Brazil	Retrospective observational, 31 patients	PE did not increase hospital mortality and showed wide variability in time to death.
E20	Fehnel et al., 2020^(31)^. USA	Retrospective observational, 822 patients	Early administration of opioids reduces the risk of severe tachypnea, especially in patients with greater clinical severity.
E21	Efstathiou et al., 2020^(32)^. Canada	Systematic review of mixed methods, 25 studies	There is great variability in practices, with terminal weaning being the most common approach, while terminal extubation is more frequent in North America and associated with greater intensity of respiratory symptoms.
E22	Robert et al., 2020^(33)^. France	Prospective observational study, 450 patients	Deep sedation and the use of vasopressors were associated with less discomfort after withdrawal of mechanical ventilation.
E23	Yeh et al., 2019^(34)^. USA	Retrospective observational, 38 patients	High frequency of distress after PE indicates need for more effective sedation.
E24	Janssens, 2017^(35)^. France	Prospective observational, 458 patients and relatives.	Immediate extubation caused more respiratory distress than terminal weaning.
E25	Rebelatto and Moritz, 2017^(36)^. Brazil.	Retrospective observational study, 23 patients	Survival after PE was related to the severity of the disease, not to the procedure itself.
E26	Robert et al., 2017^(7)^. France	Prospective observational study, 458 patients undergoing PE, their families and professionals	Immediate extubation did not alter the psychological well-being of family members, but was associated with greater respiratory distress in patients compared to terminal weaning.
E27	Thellier et al., 2017^(37)^. France	Retrospective observational study, 68 patients	Immediate extubation was associated with a shorter time to death, while terminal weaning was better accepted by nursing staff.
E28	Cottereau et al., 2016^(38)^. France	Cross-sectional, 451 health professionals (nurses and doctors)	Preference for terminal weaning was associated with lack of knowledge or unfavorable perception of PE.
E29	Delaney and Downar, 2016^(39)^. Canada	Integrative review, 99 studies	Great variability in the withdrawal of life support due to the scarcity of robust evidence.
E30	Graeff et al., 2016^(40)^. Netherlands	Literature review, does not describe	There is a shortage of structured protocols for end-of-life care in the ICU.
E31	Epker et al., 2015^(41)^. Netherlands.	Prospective observational study, 241 patients	Progressive use of opioids and sedatives was associated with rapid death and little discomfort.
E32	Long et al., 2015^(42)^. USA.	Retrospective observational, 330 patients	The majority of patients died within an hour or up to 24 hours after mechanical ventilation was withdrawn, with the shorter time to death being associated with the presence of diabetes and higher positive end-expiratory pressure values.
E33	Campbell et al., 2015^(43)^. USA.	Almost experimental, 14 patients	Nurse-led algorithm improved respiratory comfort during PE.
E34	Huynh et al., 2013^(44)^. USA.	Retrospective observational, 322 patients	PE was more frequent in older patients and with a record of expected death, and was less common in surgical services; the shorter time to death was associated with the use of vasopressors and high fractions of inspired oxygen.
E35	Epker et al., 2011^(45)^. Netherlands	Retrospective observational study, 80 patients	The combined withdrawal of ventilation and vasoactive drugs reduced the time to death.
E36	Mazer et al., 2011^(46)^. USA	Prospective observational study, 74 patients	Higher palliative doses of opioids were associated with longer time to death.
E37	Cooke et al., 2010^(47)^. USA.	Retrospective observational study, 1,505 patients	Demographic variables and measures of underlying disease severity partially explain time to death after PE.
E38	Kompanje et al., 2008^(48)^. USA.	Literature review, does not describe	Phased protocol favored symptom control and peaceful death.
E39	Fartoukh et al., 2005^(49)^. France	Retrospective observational, 5 patients	Terminal extubation proved to be viable and transparent in patients with irreversible neurological damage, with death occurring within three days of the procedure.
E40	Rocker et al., 2004^(50)^. Canada.	Prospective observational study, 206 patients	Most patients died after extubation, with the predominant use of morphine, midazolam and lorazepam, and relatives reported comfort regardless of technique or time to death.
E41	Chan et al., 2004^(51)^. USA.	Retrospective observational, 75 patients	Narcotics and benzodiazepines did not accelerate death after PE.
E42	Cook et al., 2003^(52)^ Canada, USA, Sweden, Australia.	Prospective observational study, 851 patients	Withdrawal of life support was mainly influenced by clinical medical judgment and perceived poor survival, reinforcing the importance of considering patient preferences at the end of life.
E43	Kirchhoff et al., 2003^(53)^. USA	Descriptive, with a quantitative approach, 31 Nurses	Nurses play a central role in preparing families for ventilator withdrawal.
E44	Mayer and Kossoff, 1999^(54)^. USA	Retrospective observational, 74 patients	Terminal extubation was performed in 43% of patients, with variable survival, frequent respiratory signs and predominant use of opioids to relieve discomfort, and the process was well accepted by family members.

As for the location of the studies, most were conducted in the United States (US), with 21 studies (47.7%), followed by France, with six (13.6%), and Brazil, with five (11.4%). Canada and the Netherlands each had three studies (6.8%), while Austria, India, Taiwan, and Spain each contributed one study (2.3%). It should be noted that the percentages referring to the country where the studies were conducted are not mutually exclusive, since some studies were conducted in more than one country. In addition, two studies (4.5%) were conducted in different countries, and overall, four (9.1%) were multicenter studies.

Regarding the study design, observational studies predominated, with a total of 25 publications (56.8%), of which 13 (29.5%) were retrospective cohort studies, 10 (22.7%) were prospective cohort studies, and two (4.5%) were cross-sectional studies. Next, we highlight the review studies, which correspond to 13 publications (29.5%). Three descriptive studies (6.8%) were also identified, and finally, one case report, one quasi-experimental study, and one intervention study, each representing 2.3% of the sample. The main methodological characteristics of the articles analyzed are presented in Chart 3.

The evidence summarized in Chart 4 was analyzed using the PAGER methodology, allowing for a structured understanding of the advances and gaps in scientific production on PE in the ICU. Seven main patterns were identified: recommendations for practice in palliative extubation (14 studies), variability in time to death after palliative extubation (11 studies), comparison between terminal extubation and terminal weaning (6 studies), symptoms during palliative extubation (5 studies), experiences, challenges and support for professionals (5 studies), implementation of protocols (4 studies) and determinants of the decision for palliative extubation (3 studies). These findings highlight the complexity of the palliative extubation process and the diversity of clinical, ethical and organizational dimensions addressed in the literature.

**Chart 4:** Synthesis of knowledge guided by the PAGER framework – Natal, RN, Brazil, 2025.

Standards	Advances	Gaps	Evidence for practice	Recommendations for research
Recommendations for Practice in Palliative Extubation (E1, E2, E9, E10, E11, E16, E18, E21, E29, E30, E33, E38, E39, E43).	Growing recognition of the need for structured planning, adequate symptom management, clear communication with family members and coordinated interprofessional work.	High variability between institutions and lack of robust, standardized clinical guidelines for the management of PE in the ICU.	Evidence-based practices - such as systematic symptomatic assessment, communication protocols and team support - reduce conflict, increase safety and improve the patient and family experience.	Conduct robust multicentre studies to support the development of standardized clinical guidelines applicable to different institutional contexts.
Variability in time to death after Palliative Extubation (E8, E13, E25, E32, E34, E35, E36, E37, E40, E41, E44).	Most patients die within 24 hours of PE, despite individual variability. Predictors of faster death include high APACHE II, vasopressors, high FiO2, hemodynamic instability and respiratory compromise. Opioids and proportional sedatives do not accelerate death.	Methodological heterogeneity, limited samples and clinical differences make it difficult to generalize the findings. Insufficient studies on cultural and organizational factors that may influence survival after PE.	Time to death depends on pre-extubation clinical severity, and not on analgesia or sedation. Predictors of early death help with care planning, team preparation and realistic communication with family members.	Develop and validate multicenter prognostic models to estimate time to death after PE. Expand studies in different contexts to better understand variability and improve communication with families about the end-of-life process.
Terminal extubation vs. terminal weaning (E3, E4, E24, E26, E27, E28).	Immediate extubation is associated with a higher frequency of respiratory events and a shorter time to death; terminal weaning is perceived as less abrupt and more acceptable by the team.	There is a lack of understanding of the factors that guide the choice between techniques, including cultural, ethical and institutional influences and family perceptions.	Both approaches are ethically acceptable and should be selected according to clinical condition, patient preferences and local protocols.	Develop high-quality comparative studies that evaluate clinical outcomes, family members’ experience and professionals’ perceptions.
Symptoms during Palliative Extubation (E20, E22, E23, E31, E44).	Respiratory distress is frequent, but can be controlled with the early use of opioids and proportional sedation, ensuring adequate relief of suffering.	Scarcity of standardized methods for measuring symptoms and evaluating the effectiveness of interventions pharmacological.	The ethical use of sedatives and analgesics to relieve suffering is justified, even if it may secondarily shorten life (principle of double effect).	Carry out multicenter studies using validated instruments, such as the RDOS, and promote their cultural and linguistic adaptation for different countries.
Experiences, challenges and support for professionals (E6, E10, E12, E14, E15).	There is growing recognition of the emotional, ethical and organizational burden of PE, with emphasis on the importance of integrated teams, interdisciplinary communication and mutual support.	There are few quantitative studies on the emotional, physiological and occupational impact, as well as few evaluated interventions to reduce staff suffering.	Psychological support, technical training and monitoring by experienced professionals reduce the emotional impact; debriefing and structured protocols increase safety and reduce conflicts.	Developing longitudinal studies and testing structured interventions (simulation, CP training, psychological and educational support) aimed at mental health and team performance.
Implementation of protocols (E5, E6, E17, E38).	Institutional protocols can reduce conflicts, clarify conduct and provide greater safety for patients, families and professionals.	Little availability of validated protocols and low institutional adherence, even when guidelines are in place	Well-structured protocols promote uniformity, quality of care and comfort during the PE process.	Develop, validate and implement multidisciplinary protocols adapted to cultural and institutional realities.
Determinants of the decision for Palliative Extubation (E7, E34, E42).	Decisions are influenced by clinical factors, institutional context, staff perception and patient preferences; greater chance of PE in patients with less ventilatory support and expected death.	Lack of uniform criteria and little understanding of the cultural, institutional and subjective variations that shape the decision.	The decision should integrate clinical assessment, patient preferences and structured communication with relatives and staff, favoring shared decisions.	Invest in multicenter studies that compare practices between institutions and develop objective criteria and tools to support ethical decisions at the end of life.

## DISCUSSION

When analyzing the set of studies included, there has been a significant increase in research on PE in recent years. This growth is directly related to the expansion of the use of life support therapies in ICUs and the need to reassess LSL in irreversible situations. Thus, recent scientific production reflects a growing demand for theoretical and practical references to guide complex clinical decisions and promote ethical, safe, and humanized end-of-life care.

Despite this quantitative advance, the literature still lacks robust evidence to support the standardization of PE practice. Observational studies predominate, which limits the generalization of findings and highlights the need for investigations with greater methodological rigor, such as clinical trials and intervention studies. This limitation is particularly relevant, considering that PE involves decisions with a high impact on patients, family members, and multidisciplinary teams.

Based on the analysis of the evidence gathered, scientific production on PE was organized into different analytical patterns, which highlight both advances and gaps in the understanding of this practice. Among the patterns identified, recommendations for clinical practice in PE stand out. Studies converge on the need for a structured process involving transparent communication, rigorous symptom management, and continuous support for patients, families, and teams^(3,10,18,21,26)^. The findings reinforce the central role of nurses in coordinating the procedure, providing emotional support, and promoting a humanized care environment^(22,29)^.

In addition, the importance of detailed planning after the decision for PE is evident. This planning should include interdisciplinary and family meetings, definition of the method (terminal weaning or immediate extubation), prior care, drug management strategies, and subsequent care^(55)^. Preparation involves setting the date and time, organizing the multidisciplinary team, documentation, adapting the environment, and providing guidance to the patient and family; subsequent care includes maintaining sedation, monitoring symptoms, possible patient transfer, and support for teams and family members^(35,55)^.

Inadequate management of PE can expose patients to significant complications, especially respiratory distress, one of the most difficult symptoms to manage and particularly frequent^(33,43)^. In addition to the direct impact on the patient, this suffering has a significant impact on family members and the multidisciplinary team, increasing the emotional burden and complexity of care^(27)^.

In this context, it is clear that PE affects not only those receiving care, but also those providing it: studies show that healthcare professionals often experience emotional distress, exhaustion, and persistent grief when managing the dying process, often without adequate institutional support^(25,26)^. Given this, it is recommended to adopt structured support strategies, such as peer support, reflective groups, psychological and spiritual resources, as well as formal debriefings after each procedure - in order to mitigate emotional distress and improve the quality of care^(10,25)^.

The literature also shows that the technique used in PE directly influences the expression of symptoms, which reinforces the need for adequate team preparation. French studies have observed that immediate extubation was associated with gasping, bronchial obstruction, and pain, effects that were attenuated when deep sedation was used^(33)^. In the Netherlands, the proportional use of opioids and sedatives kept the incidence of respiratory distress low without anticipating death, showing that adequate symptomatic management is safe and essential to ensure a dignified death^(41)^. These findings converge in indicating that the fear of shortening life should not restrict the use of pharmacological measures, at the risk of undertreatment and avoidable suffering^(10)^.

Brazilian studies show wide variability in the interval to death after PE: in one of them^(30)^, most patients died after 24 hours, with a time ranging from 1 to 19 days; in another^(36)^, an average of 2.5 days was identified between extubation and death. In line with this heterogeneity, international evidence indicates that the time to death is determined mainly by clinical severity and pre-extubation physiological parameters—such as elevated APACHE II, hemodynamic instability, use of vasopressors, and need for high FiO_2_—rather than by the use of opioids or sedatives. Thus, the immediate outcome mainly reflects the patient’s baseline condition, regardless of the technique applied. Although survival varies widely, most patients die within 24 hours after PE, reinforcing the role of previous clinical markers as the main predictors of time to death^(20,42)^.

From an ethical standpoint, there is consensus that proportional sedation and the use of analgesics are morally justifiable when guided by the relief of suffering. Even though such interventions may secondarily shorten life, their primary purpose—to control dyspnea and other discomforts—legitimizes them, provided they are applied with clear and documented intent^(33)^.

To advance the quality of care and with the aim of assisting healthcare professionals in assessing the need for medication titration to relieve discomfort during PE in end-of-life care, Margaret L. Campbell developed the Respiratory Distress Observation Scale (RDOS). This scale measures the presence and intensity of respiratory distress in patients who are unable to report the sensation of dyspnea. Each parameter is assessed with a score from 0 to 2, and the scores are added to obtain a total value ranging from 0, indicating no suffering, to 16, representing intense respiratory distress^(27)^. However, the lack of cross-cultural validation of this scale for Brazilian Portuguese limits its applicability in national practice.

Regarding methods of discontinuing mechanical ventilation, there is no evidence to indicate superiority between immediate extubation and terminal weaning. Immediate extubation tends to be perceived as a more natural process, although it is associated with a higher risk of respiratory distress^(7,55)^. Terminal weaning, on the other hand, is considered less abrupt and generally more aligned with the preferences of nurses, nursing technicians, and physicians. This is evidenced in a recent study that points to a marked predominance of the choice for terminal weaning among physicians, being adopted by 90% of professionals, while only one opted for immediate extubation^(56)^.

The decision between terminal weaning and immediate extubation should consider the level of ventilatory support, the risk of symptoms after withdrawal of support, and the preferences of the patient and family. Terminal weaning is more appropriate when the patient depends on moderate to high ventilatory support, allowing for progressive reduction and better comfort management. Immediate extubation, on the other hand, tends to be appropriate in scenarios of low support and low probability of significant discomfort. Regardless of the method chosen, prior optimization of sedation and analgesia remains a fundamental requirement to ensure comfort and dignity in the palliative extubation process^(10)^.

The findings of this review show wide variability in the conduct of PE, both between countries and within the same healthcare system, influenced by institutional differences, clinical experience, professional values, family preferences, and cultural, religious, and legal factors. This heterogeneity stems not only from the clinical conditions of patients, but above all from the institutional context. A multicenter study showed that the probability of PE is more determined by the center where care is provided than by physiological parameters, with significantly different rates between countries, indicating the predominance of local practices and cultures over individualized decision-making^(19)^.

Similarly, a study demonstrated wide global variability in LSL practices, including the withdrawal of devices such as in PE. The differences were significant between regions, with a greater predominance of abstinence or withdrawal of therapies in Northern European countries and Australia/New Zealand, while Latin America and Africa had a higher frequency of deaths after failed cardiopulmonary resuscitation. These patterns reflect cultural, institutional, and legal influences and distinct decision-making models, highlighting that practices such as PE are deeply shaped by context^(57)^.

Although clinical factors, such as lower ventilatory support and expectation of death, increase the likelihood of PE, the preponderance of the institutional environment demonstrates that the lack of standardization compromises the predictability and consistency of care, which can generate clinical uncertainty, ethical conflicts, and greater family distress^(19)^.

In this scenario, it is essential to adopt clear, evidence-based protocols to reduce variability in care and promote more consistent conduct. Structured protocols favor multidisciplinary communication, guide procedure planning, and ensure that decisions are aligned with the values of the patient and family, in addition to minimizing conflicts and reducing the emotional distress of the team^(10,17,18)^. Thus, standardization is a central element in qualifying the practice of PE, strengthening the safety, humanization, and technical robustness of end-of-life care.

Given this scenario, PE is a complex procedure, permeated by clinical, ethical, and sociocultural dimensions. This complexity requires ICU professionals to receive specific training, covering not only technical aspects but also communication skills, conflict management, and emotional support, ensuring quality and safety at all stages of the process.

The main limitation of this review was the exclusion of restricted-access articles and the scarcity of studies with a high level of evidence, especially in the national context, which reflects the difficulties of conducting research at the end of life. In addition, the focus on adults in the ICU restricts the generalization of the findings, since the literature points to experiences in pediatric and home settings.

Nevertheless, this review contributes significantly to clinical practice, teaching, and research in palliative care, health, and nursing. For academic training, it points to the need for innovative pedagogical strategies that prepare future professionals to deal with the complexity of PE. In the scientific field, it highlights research priorities, such as the comparison between PPE methods, the adaptation of assessment instruments, the analysis of the emotional impact on professionals, and the development of validated protocols that consider the cultural and care specificities of each context.

For healthcare professionals, it offers support for ethical and safe decision-making, guiding symptom management, communication with family members, and multidisciplinary integration. Specifically, with regard to nursing professionals, it reinforces the importance of the role of this category in symptom management, support for family members, and multidisciplinary communication, improving end-of-life care.

Therefore, the findings of this review reinforce the need to strengthen the scientific basis that guides PE, through the production of robust evidence and the implementation of training and organizational strategies that ensure quality, ethics, and humanization in end-of-life care.

## CONCLUSION

The scope review showed that PE in the ICU is a complex practice, permeated by clinical, ethical, and organizational challenges. Consistent patterns were identified related to symptom management, communication with family members, multidisciplinary integration, and advance care planning, showing that the adoption of structured practices can improve end-of-life care.

However, there was considerable variability in the practice of PE, a lack of validated protocols, and a scarcity of robust studies, especially on the comparison between terminal extubation and terminal weaning. The choice of method was influenced by clinical, cultural, and institutional factors, highlighting the need for uniform, evidence-based criteria.

Although appropriate pharmacological management has been shown to be effective in controlling respiratory distress without hastening death, the emotional impact of PE on professionals remains understudied, indicating an important field for research. Thus, we recommend the development of comparative clinical trials, the expansion of studies in different care contexts, the development of validated multidisciplinary protocols, the cross-cultural adaptation of discomfort assessment instruments, and the analysis of technical and psychological support strategies for teams.

Advances in knowledge about PE have significant potential to improve end-of-life care in intensive care, contributing to safer, more ethical practices that are aligned with the needs of patients and their families.

## Data Availability

The entire dataset supporting the results of this study was published in the article itself.
