# Supplementary material for: Palliative extubation in intensive care units: scoping review
Source: Rev Esc Enferm USP. 2026 Mar 16;60:e20250409. doi: 10.1590/1980-220X-REEUSP-2025-0409en (PMC12994597; doi:10.1590/1980-220X-REEUSP-2025-0409en)
Supplement: Translation

# Translation: Extubação paliativa em unidades de terapia intensiva: revisão de escopo

**DOI:** 10.1590/1980-220X-REEUSP-2025-0409pt

**Authors:** Rafaella Guilherme Gonçalves, Lucas Batista Ferreira, Tarcísio Tércio das Neves, Carlos Jordão de Assis Silva, Fabiane Rocha Botarelli, Rejane Maria Paiva de Menezes

**Affiliations:** 1 Universidade Federal do Rio Grande do Norte, Departamento de Enfermagem, Natal, RN, Brasil.

**Keywords:** Cuidados Paliativos, Unidades de Terapia Intensiva, Equipe de Assistência ao Paciente, Extubação, Enfermagem

## Abstract

**Objetivo:**

Mapear o conhecimento existente sobre a extubação paliativa em pacientes adultos internados em unidades de terapia intensiva.

**Método:**

Revisão de escopo conduzida conforme as diretrizes do JBI. As buscas ocorreram entre outubro e dezembro de 2024, em sete bases de dados e quatro repositórios, além de busca reversa. A seleção foi independente, a extração seguiu protocolo estruturado e o relato utilizou PRISMA-ScR. A análise dos resultados baseou-se no framework PAGER (*Patterns, Advances, Gaps, Evidence for practice and Research recommendations*).

**Resultados:**

A busca identificou 1.463 materiais e incluiu 44 estudos. Predominaram as publicações no ano de 2023. Os achados abordaram recomendações para a prática, tempo até o óbito, comparação entre extubação terminal e desmame terminal, manejo de sintomas, vivências e desafios dos profissionais, além de aspectos relacionados à tomada de decisão e ao uso de protocolos na extubação paliativa.

**Conclusão:**

A extubação paliativa permanece marcada por práticas heterogêneas e e produção limitada de evidências de alto nível. Há necessidade de protocolos baseados em evidências e capacitação multiprofissional para garantir cuidado de qualidade, dignidade e alívio do sofrimento no fim da vida.

## INTRODUÇÃO

Pacientes com doenças ou condições que ameaçam a vida podem evoluir para quadros críticos, caracterizados por instabilidade ou risco iminente de falência orgânica e morte. Nessas situações, a assistência costuma ocorrer em Unidades de Terapia Intensiva (UTI) ou de Cuidados Intermediários (UCI), ambientes preparados para oferecer suporte vital de alta complexidade, com monitorização contínua e recursos voltados à manutenção da vida^(1)^. Apesar dos avanços tecnológicos, a mortalidade nas UTI brasileiras permanece elevada, variando entre 9,6% e 58%, influenciada por fatores como gravidade clínica, comorbidades, tempo de internação, ventilação mecânica, sepse, insuficiência renal aguda e qualidade da assistência^(2)^.

A integração precoce dos cuidados paliativos (CP) ao tratamento modificador da doença é essencial para o cuidado integral do paciente crítico, contribuindo para evitar intervenções fúteis, reduzir tempo de internação e custos, aliviar o sofrimento e preservar a dignidade, sem antecipar o processo de morrer^(3)^. Quando incorporados de forma concomitante, os CP favorecem decisões éticas e humanizadas, com foco no controle rigoroso dos sintomas^(2)^.

Além disso, a introdução precoce dos CP favorece a definição dos Limites de Suporte de Vida (LSV), permitindo que profissionais e familiares participem das decisões sobre o plano terapêutico e acompanhem a progressão dos CP até a fase de fim de vida^(4)^. O LSV consiste em não iniciar, suspender ou limitar intervenções de suporte vital em situações irreversíveis, evitando tratamentos fúteis que prolonguem artificialmente o processo de morrer^(5)^. No ambiente da UTI, a discussão é particularmente relevante, incluindo decisões sobre hemodiálise, drogas vasoativas, nutrição artificial e ventilação mecânica invasiva, sendo esta última, geralmente, a medida final a ser descontinuada, pois sua retirada costuma determinar o óbito em curto prazo^(6,7)^.

A extubação paliativa (EP) refere-se à retirada planejada da ventilação mecânica em pacientes com morte iminente, sendo reconhecida como uma prática ética e moralmente justificável por evitar a distanásia e permitir a morte com conforto e dignidade^(8)^. Esse procedimento exige preparo técnico, comunicação efetiva e atuação multiprofissional qualificada, de modo a assegurar o controle de sintomas e o suporte adequado ao paciente e à família^(9)^.

Entretanto, a implementação da EP ainda enfrenta desafios significativos. Entre eles, destacam-se barreiras culturais, religiosas e emocionais, a ausência de protocolos institucionais claros e a heterogeneidade de condutas entre equipes^(10)^.

No contexto brasileiro, as dificuldades são agravadas por lacunas na formação acadêmica e profissional sobre o processo de morrer e morte, pela insuficiente capacitação em comunicação de más notícias e pelas limitações estruturais para integração dos CP na terapia intensiva^(5)^. Assim, a EP se configura como uma das condutas de LSV mais complexas e sensíveis, exigindo preparo técnico, ético e emocional das equipes de saúde.

Diante desse cenário, torna-se fundamental ampliar o debate e as reflexões sobre a atuação dos profissionais de saúde no manejo adequado da EP, com especial atenção à Enfermagem, que desempenha papel central na promoção do conforto físico, psíquico, social e espiritual dos pacientes em fim de vida. Assim,este estudo tem como objetivo mapear o conhecimento existente sobre a extubação paliativa em pacientes adultos internados em unidades de terapia intensiva, sintetizando os achados segundo o *framework* PAGER (*Patterns, Advances, Gaps, Evidence for practice and Research recommendations*).

## MÉTODO

### Desenho do Estudo

Trata-se de revisão de escopo, que buscou explorar, mapear, sintetizar evidências científicas e identificar as lacunas de conhecimento acerca da EP em pacientes adultos internados em UTI. O método seguiu as orientações do JBI para revisões de escopo^(11)^ e a redação atendeu às recomendações do PRISMA-ScR^(12)^. O protocolo dessa revisão foi cadastrado no Open Science Framework (DOI: 10.17605/OSF.IO/JYMNZ).

### Coleta de Dados

Inicialmente, realizou-se uma busca preliminar nas bases MEDLINE e Scopus com o objetivo de identificar estudos relacionados ao tema, bem como selecionar palavras-chave e termos de índice utilizados nessas publicações. Para a elaboração da questão norteadora, adotou-se o acrônimo PCC, descrito no Quadro 1.

**Quadro 1:** Estratégia PCC (População, Conceito e Contexto) – Natal, RN, Brasil, 2025.

Acrônimo	Termo	Descrição
P	População	Pacientes adultos em cuidados de fim de vida
C	Conceito	Extubação paliativa
C	Contexto	Unidades de terapia intensiva

A partir dessa estratégia, formulou-se a seguinte questão de pesquisa: “Qual o conhecimento existente na literatura sobre extubação paliativa de pacientes adultos internados em unidades de terapia intensiva?”

O levantamento bibliográfico ocorreu entre outubro e dezembro de 2024 por meio do portal de periódicos da Coordenação de Aperfeiçoamento de Pessoal de Nível Superior (CAPES), via Comunidade Acadêmica Federada (CAFe), contemplando as bases SCOPUS (Elsevier), MEDLINE/PubMed (National Library of Medicine), *Web of Science*, CINAHL (*Cumulative Index to Nursing and Allied Health Literature*), *Google Scholar*, LILACS (Literatura Latino-Americana e do Caribe em Ciências da Saúde) e BDENF (Base de Dados da Enfermagem). Complementarmente, realizou-se busca reversa nas referências dos artigos incluídos, a fim de identificar estudos adicionais pertinentes. Para ampliar o escopo, também foram consultadas fontes de literatura cinzenta, incluindo teses e dissertações disponíveis em repositórios online: Catálogo de Teses e Dissertações da CAPES, BASE (*Bielefeld Academic Search Engine*), *Cybertesis* e *Open Access Theses & Dissertations* (OATD).

Os descritores selecionados para realizar a busca foram os termos do Descritores em Ciências da Saúde (DeCS) e os correspondentes no *Medical Subject Headings* (MESH): Cuidados Paliativos/Palliative Care, Extubação/Airway Extubation e Unidades de Terapia Intensiva/Intensive Care Units, além das Keywords: Terminal Care, End-of-life care, Compassionate Extubation, Terminal weaning e Terminal extubation e da palavra-chave: Extubação Paliativa.

As estratégias de busca foram elaboradas e adaptadas de acordo com as especificidades sintáticas e operacionais de cada base de dados, respeitando os respectivos campos de indexação, o uso de operadores booleanos e os recursos próprios de recuperação da informação. O Quadro 2 apresenta as estratégias de busca adotadas em cada base de dados.

**Quadro 2:** Estratégia de busca utilizada para cada base de dados – Natal, RN, Brasil, 2025.

Base	Estratégia
SCOPUS	(“Palliative Care” OR “Terminal Care” OR “End-of-life Care”) AND (“Airway Extubation” OR “Compassionate Extubation” OR “Terminal Extubation” OR “Terminal Weaning”) AND (“Intensive Care Units”)
MEDLINE/PubMed	(Palliative Care OR Terminal Care OR End-of-life Care) AND (Airway Extubation OR Compassionate Extubation OR Terminal Extubation OR Terminal Weaning) AND (Intensive Care Units)
Web of Science	ALL=(Palliative Care OR Terminal Care OR End-of-life Care) AND ALL=(Airway Extubation OR Compassionate Extubation OR Terminal Extubation OR Terminal Weaning) AND ALL=(Intensive Care Units)
CINAHL	(Palliative Care OR Terminal Care OR End-of-life Care) AND (Airway Extubation OR Compassionate Extubation OR Terminal Extubation OR Terminal Weaning) AND (Intensive Care Units)
Google Scholar	(“Palliative Care” OR “Terminal Care” OR “End-of-life Care”) AND (“Airway Extubation” OR “Compassionate Extubation” OR “Terminal Extubation” OR “Terminal Weaning”) AND (“Intensive Care Units”)
LILACS	Palliative Care AND Airway Extubation
BDENF	Palliative Care AND Airway Extubation
**Literatura Cinzenta**	**Estratégia**
Catálogo de teses e dissertações da CAPES	Extubação Paliativa
BASE	Palliative Care AND Airway Extubation
Cybertesis	Palliative Care AND Airway Extubation
OATD	Palliative Care AND Airway Extubation AND Intensive Care Units

### Critérios de Seleção

Os critérios de seleção foram estabelecidos com base na questão norteadora, considerando a estratégia PCC. Foram considerados estudos com as seguintes características: a) quanto à população – estudos realizados com pacientes em cuidados de fim de vida, com idade de 18 anos ou superior; b) quanto ao conceito – estudos que avaliaram ou descreveram a realização da EP; c) quanto ao contexto – estudos que contemplem a EP realizada nas UTI. Além disso, foram incluídos estudos de pesquisas primárias com delineamentos quantitativos, qualitativos e de métodos mistos, estudos de revisão e de opinião clínica, disponíveis na íntegra, sem restrição de idioma e sem recorte temporal. Foram excluídos resumos e cartas ao editor.

### Extração e Análise dos Dados

A seleção dos estudos foi conduzida por dois revisores, de forma independente e cegada, em duas fases: triagem de títulos/resumos e leitura na íntegra dos textos potencialmente elegíveis. Duplicatas foram removidas e, em caso de discordância, um terceiro revisor foi consultado. Adicionalmente, realizou-se busca reversa nas referências dos estudos incluídos. O processo de identificação, triagem e inclusão está detalhado em fluxograma conforme diretriz PRISMA-ScR^(12)^.

Para a extração dos dados, elaborou-se um instrumento de coleta contendo as variáveis de interesse: autores, ano de publicação, país, objetivos, método, população e tamanho amostral, desfechos e principais resultados relacionados à questão da revisão. As informações extraídas foram organizadas em planilha eletrônica e apresentadas por meio de estatística descritiva. Além disso, elaborou-se um quadro-resumo para facilitar a visualização e síntese dos achados.

Quanto a análise, utilizou-se a metodologia PAGER (Patterns, Advances, Gaps, Evidence for practice and Research recommendations), a qual amplia a qualidade e o rigor das revisões de escopo por meio de uma análise estruturada dos achados. Essa abordagem contempla: (1) Padrões – principais temas emergentes; (2) Avanços – novas descobertas, insights teóricos ou metodológicos; (3) Lacunas – áreas que demandam investigações adicionais; (4) Evidências para a prática – achados relevantes para aplicação prática; e (5) Recomendações de pesquisa – direções para estudos futuros^(13)^.

### Aspectos Éticos

A pesquisa foi conduzida conforme os princípios éticos e metodológicos aplicáveis, com respeito às boas práticas científicas e aos direitos autorais das obras consultadas. Por tratar-se de revisão baseada em dados secundários de acesso público, sem envolvimento direto de seres humanos, não houve necessidade de submissão ao comitê de ética em pesquisa.

## RESULTADOS

A busca realizada entre outubro e dezembro de 2024 identificou 1.463 registros, sendo 1.389 provenientes das bases de dados e 74 da literatura cinzenta. Após a triagem de títulos e resumos, 1.389 estudos foram excluídos por não atenderem aos critérios de elegibilidade, restando 74 para leitura em texto completo. Na etapa seguinte, foram removidas 26 duplicatas (23 das bases e 3 da literatura cinzenta), totalizando 48 estudos elegíveis. Destes, oito foram excluídos por não apresentarem o cenário de realização ou por se distanciarem do tema proposto. Após a leitura integral, 40 estudos foram incluídos, acrescidos de quatro identificados por busca manual nas referências. Assim, a amostra final compreendeu 44 estudos, conforme ilustrado no fluxograma PRISMA-ScR (Figura 1).

**Figura 1:** Fluxograma PRISMA-ScR de seleção das publicações – Natal, RN, Brasil, 2025.

Os 44 estudos incluídos nesta revisão de escopo consistem em publicações dos últimos 25 anos, compreendidos entre 1999 e 2024, com predomínio de sete estudos (15,9%) publicados em 2023. Além disso, 2024 já se apresenta como um ano promissor para pesquisas sobre a temática, tendo sido identificadas seis publicações (13,6%) realizadas nesse período. Essas informações estão apresentadas no Quadro 3.

**Quadro 3:** Caracterização dos estudos quanto ao autor, ano, país de origem, objetivo, desenho do estudo, população e principais resultados – Natal, RN, Brasil, 2025.

Id	Autores/Ano/País	Tipo de estudo, população e amostra	Principais resultados
E1	Jennerich, 2024^(3)^. EUA	Revisão da literatura, 83 estudos	Controle adequado de sintomas e preparo da equipe são essenciais para minimizar sofrimento durante e após a EP.
E2	Schaden et al., 2024^(14)^. Áustria	Revisão da literatura, não descreve	Transição precoce para cuidados de conforto favorece dignidade no fim da vida; EP requer preparo rigoroso.
E3	Kumar et al., 2024^(15)^. Índia	Revisão da literatura, 15 estudos	EP e desmame terminal são opções éticas quando alinhadas aos interesses do paciente terminal.
E4	Curtisi et al., 2024^(16)^. EUA	Relato de caso, 2 pacientes	O desmame gradual associado ao manejo adequado de sintomas proporcionou conforto, enquanto a extubação abrupta com medicação insuficiente resultou em sofrimento.
E5	Marica, 2024^(17)^. EUA	Estudo de Intervenção	Uma diretriz multidisciplinar e baseada em evidências para EP aumenta segurança profissional e qualidade do cuidado.
E6	Bryan et al., 2024^(18)^. EUA	Observacional prospectivo, com abordagem qualitativa, 312 profissionais de saúde	Comunicação e integração multiprofissional, aliadas ao uso de protocolos, são fundamentais para uma EP segura e humanizada, sendo a relação equipe–família central no processo decisório de fim de vida.
E7	Waldauf et al., 2023^(19)^. Canadá, Tchéquia e Holanda	Observacional prospectivo, 616 pacientes	A decisão pela EP foi influenciada pelo contexto institucional e associada a menor suporte ventilatório e menor sobrevida.
E8	Zheng et al., 2023^(20)^. Taiwan	Observacional retrospectivo, 140 pacientes	O tempo até o óbito após a EP foi variável, sendo menor em pacientes com maior gravidade clínica, o que auxilia no planejamento dos cuidados paliativos.
E9	Mazzu et al., 2023^(21)^. EUA	Revisão da literatura, 73 estudos	Comunicação estruturada e suporte à equipe são práticas-chave; evidências robustas ainda são limitadas.
E10	Ortega-Chen et al., 2023^(10)^. EUA	Revisão da literatura, não descreve	EP é um processo complexo e variável, exigindo habilidades paliativas de comunicação, planejamento cuidadoso, avaliação e gerenciamento de sintomas e debriefing.
E11	Roberts e Dahlin, 2023^(22)^. EUA	Revisão narrativa, não descreve	Enfermeiros têm papel central e podem liderar práticas baseadas em evidências na EP.
E12	Bastos et al., 2023^(23)^. Brasil	Descritivo, com abordagem qualitativa, profissionais de saúde	Profissionais reconhecem benefícios da EP, mas relatam insegurança técnica e necessidade de capacitação.
E13	Antonio e Antonio, 2023^(24)^. Brasil	Observacional prospectivo, 18 pacientes	Implementação de protocolo local organizou a prática e evidenciou variabilidade no tempo até o óbito.
E14	Kaur et al., 2022^(25)^. EUA	Transversal, 20 profissionais de saúde	A EP associou-se a sofrimento emocional significativo entre profissionais de saúde, especialmente entre mulheres jovens e aqueles insatisfeitos com o processo institucional.
E15	Orr et al., 2022^(26)^ EUA	Descritivo, com abordagem qualitativa, 20 profissionais da saúde – médicos, enfermeiros e fisioterapeutas.	Experiências profissionais influenciam a condução da EP; treinamento e suporte reduzem conflitos.
E16	Cooney-Newton e Hare, 2022^(27)^. EUA	Revisão da literatura, não descreve	A Escala de Observação do Desconforto Respiratório é uma ferramenta objetiva que auxilia na titulação de medicamentos para conforto durante os cuidados de fim de vida e a EP.
E17	Correa-Pérez, 2022^(28)^. Espanha	Revisão da literatura, não descreve	Protocolos institucionais são necessários para sedação, analgesia e apoio aos familiares.
E18	Dias, 2021^(29)^. Brasil	Revisão integrativa, 20 estudos	Enfermagem é fundamental na tomada de decisão, execução da EP e manutenção do conforto.
E19	Lacerda et al., 2020^(30)^. Brasil	Observacional retrospectivo, 31 pacientes	EP não aumentou mortalidade hospitalar e apresentou ampla variabilidade no tempo até o óbito.
E20	Fehnel et al., 2020^(31)^. EUA	Observacional retrospectivo, 822 pacientes	A administração antecipada de opioides reduz o risco de taquipneia grave, especialmente em pacientes com maior gravidade clínica.
E21	Efstathiou et al., 2020^(32)^. Canadá	Revisão sistemática de métodos mistos, 25 estudos	Há grande variabilidade nas práticas, sendo o desmame terminal a abordagem mais comum, enquanto a extubação terminal é mais frequente na América do Norte e associada a maior intensidade de sintomas respiratórios.
E22	Robert et al., 2020^(33)^. França	Observacional prospectivo, 450 pacientes	Sedação profunda e uso de vasopressores associaram-se a menor desconforto após retirada da ventilação mecânica.
E23	Yeh et al., 2019^(34)^. EUA	Observacional retrospectivo, 38 pacientes	Alta frequência de sofrimento após EP indica necessidade de sedação mais eficaz.
E24	Janssens, 2017^(35)^. França	Observacional prospectivo, 458 pacientes e familiares.	Extubação imediata causou mais desconforto respiratório que o desmame terminal.
E25	Rebelatto e Moritz, 2017^(36)^. Brasil.	Observacional retrospectivo, 23 pacientes	Sobrevida após EP relacionou-se à gravidade da doença, não ao procedimento em si.
E26	Robert et al., 2017^(7)^. França	Observacional prospectivo, 458 pacientes submetidos à EP, familiares e profissionais	A extubação imediata não alterou o bem-estar psicológico dos familiares, mas esteve associada a maior desconforto respiratório nos pacientes em comparação ao desmame terminal.
E27	Thellier et al., 2017^(37)^. França	Observacional retrospectivo, 68 pacientes	A extubação imediata associou-se a menor tempo até a morte, enquanto o desmame terminal foi melhor aceito pela enfermagem.
E28	Cottereau et al., 2016^(38)^. França	Transversal, 451 profissionais de saúde (enfermeiros e médicos)	Preferência pelo desmame terminal associou-se ao desconhecimento ou percepção desfavorável sobre EP.
E29	Delaney e Downar, 2016^(39)^. Canadá	Revisão integrativa, 99 estudos	Grande variabilidade na retirada de suporte de vida devido à escassez de evidências robustas.
E30	Graeff et al., 2016^(40)^. Holanda	Revisão da literatura, não descreve	Há escassez de protocolos estruturados para cuidados de fim de vida em UTI.
E31	Epker et al., 2015^(41)^. Holanda.	Observacional prospectivo, 241 paciente	Uso progressivo de opioides e sedativos associou-se a morte rápida e com pouco desconforto.
E32	Long et al., 2015^(42)^. EUA.	Observacional retrospectivo, 330 pacientes	A maioria dos pacientes morreu dentro de uma hora ou em até 24 horas após a retirada da ventilação mecânica, sendo o menor tempo até a morte associado à presença de diabetes e a valores mais elevados de pressão expiratória final positiva.
E33	Campbell et al., 2015^(43)^. EUA.	Quase experimental, 14 pacientes	Algoritmo conduzido por enfermeiros melhorou conforto respiratório durante a EP.
E34	Huynh et al., 2013^(44)^. EUA.	Observacional retrospectivo, 322 pacientes	A EP foi mais frequente em pacientes mais velhos e com registro de morte esperada, sendo menos comum em serviços cirúrgicos; o menor tempo até o óbito associou-se ao uso de vasopressores e a altas frações de oxigênio inspirado.
E35	Epker et al., 2011^(45)^. Holanda	Observacional retrospectivo, 80 pacientes	A retirada combinada de ventilação e drogas vasoativas reduziu o tempo até o óbito.
E36	Mazer et al., 2011^(46)^. EUA	Observacional prospectivo, 74 pacientes submetidos	Doses paliativas mais altas de opioides associaram-se a maior tempo até o óbito.
E37	Cooke et al., 2010^(47)^. EUA.	Observacional retrospectivo, 1.505 pacientes	Variáveis demográficas e medidas da gravidade da doença subjacente explicam parcialmente o tempo até a morte após EP.
E38	Kompanje et al., 2008^(48)^. EUA.	Revisão da literatura, não descreve	Protocolo em fases favoreceu controle de sintomas e morte tranquila.
E39	Fartoukh et al., 2005^(49)^. França	Observacional retrospectivo, 5 pacientes	A extubação terminal mostrou-se viável e transparente em pacientes com dano neurológico irreversível, com óbito ocorrendo em até três dias após o procedimento.
E40	Rocker et al., 2004^(50)^. Canadá.	Observacional prospectivo, 206 pacientes	A maioria dos pacientes morreu após a extubação, com uso predominante de morfina, midazolam e lorazepam, e os familiares relataram conforto independentemente da técnica ou do tempo até o óbito.
E41	Chan et al., 2004^(51)^. EUA.	Observacional retrospectivo, 75 pacientes	Narcóticos e benzodiazepínicos não aceleraram a morte após EP.
E42	Cook et al., 2003^(52)^. Canadá, EUA, Suécia, Austrália.	Observacional prospectivo, 851 pacientes	A retirada do suporte de vida foi influenciada principalmente pelo julgamento clínico médico e pela percepção de baixa sobrevida, reforçando a importância de considerar as preferências do paciente no fim de vida.
E43	Kirchhoff et al., 2003^(53)^. EUA	Descritivo, com abordagem quantitativa, 31 Enfermeiros	Enfermeiros desempenham papel central no preparo das famílias para a retirada da ventilação.
E44	Mayer e Kossoff, 1999^(54)^. EUA	Observacional retrospectivo, 74 pacientes	A extubação terminal foi realizada em 43% dos pacientes, com sobrevida variável, sinais respiratórios frequentes e uso predominante de opioides para alívio do desconforto, sendo o processo bem aceito pelos familiares.

Quanto ao local dos estudos, foram desenvolvidos majoritariamente nos Estados Unidos (EUA), com 21 estudos (47,7%), seguidos pela França, com seis (13,6%), e pelo Brasil, com cinco (11,4%). Canadá e Holanda apresentaram três estudos cada (6,8%), enquanto Áustria, Índia, Taiwan e Espanha contribuíram com um estudo cada (2,3%). Destaca-se que os percentuais referentes ao país de realização dos estudos não são mutuamente excludentes, uma vez que alguns estudos foram conduzidos em mais de um país. Além disso, dois estudos (4,5%) foram realizados em diferentes países e, no geral, quatro (9,1%) foram pesquisas multicêntricas.

Quanto ao delineamento dos estudos, predominaram os estudos observacionais, com um total de 25 publicações (56,8%), sendo 13 (29,5%) de coorte retrospectiva, 10 (22,7%) de coorte prospectiva e dois estudos transversais (4,5%). Em seguida, destacam-se os estudos de revisão, que correspondem a 13 publicações (29,5%). Também foram identificados três estudos descritivos (6,8%) e, por fim, um relato de caso, um estudo quase experimental e um estudo de intervenção, cada um representando 2,3% da amostra. As principais características metodológicas dos artigos analisados estão apresentadas no Quadro 3.

As evidências sintetizadas no Quadro 4 foram analisadas por meio da metodologia PAGER, permitindo uma compreensão estruturada dos avanços e das lacunas na produção científica sobre a EP em UTI. Foram identificados sete padrões principais: recomendações para a prática na extubação paliativa (14 estudos), variabilidade no tempo até o óbito após a extubação paliativa (11 estudos), comparação entre extubação terminal e desmame terminal (6 estudos), sintomas durante a extubação paliativa (5 estudos), vivências, desafios e suporte aos profissionais (5 estudos), implementação de protocolos (4 estudos) e determinantes da decisão pela extubação paliativa (3 estudos). Esses achados evidenciam a complexidade do processo de extubação paliativa e a diversidade de dimensões clínicas, éticas e organizacionais abordadas na literatura.

**Quadro 4:** Síntese do conhecimento guiada pela estrutura PAGER – Natal, RN, Brasil, 2025.

Padrões	Avanços	Lacunas	Evidência para a prática	Recomendações para pesquisa
Recomendações para a prática na Extubação Paliativa (E1, E2, E9, E10, E11, E16, E18, E21, E29, E30, E33, E38, E39, E43).	Reconhecimento crescente da necessidade de planejamento estruturado, manejo adequado de sintomas, comunicação clara com familiares e trabalho interprofissional coordenado.	Elevada variabilidade entre instituições e ausência de diretrizes clínicas robustas e padronizadas para a condução da EP na UTI.	Práticas baseadas em evidências - como avaliação sintomática sistemática, protocolos de comunicação e suporte à equipe - reduzem conflitos, aumentam segurança e melhoram a experiência do paciente e da família.	Conduzir estudos multicêntricos e robustos que apoiem a elaboração de diretrizes clínicas padronizadas e aplicáveis a diferentes contextos institucionais.
Variabilidade no tempo até o óbito após Extubação Paliativa (E8, E13, E25, E32, E34, E35, E36, E37, E40, E41, E44).	A maioria dos pacientes morre em até 24 horas após a EP, apesar da variabilidade individual. Preditores de morte mais rápida incluem APACHE II elevado, vasopressores, FiO2 alta, instabilidade hemodinâmica e comprometimento respiratório. Opioides e sedativos proporcionais não aceleram o óbito.	Heterogeneidade metodológica, amostras limitadas e diferenças clínicas dificultam a generalização dos achados. Insuficiência de estudos sobre fatores culturais e organizacionais que possam influenciar a sobrevida após a EP.	O tempo até o óbito depende da gravidade clínica pré-extubação, e não da analgesia ou sedação. Preditores de morte precoce auxiliam no planejamento dos cuidados, preparo da equipe e comunicação realista com familiares.	Desenvolver e validar modelos prognósticos multicêntricos para estimar o tempo até o óbito após EP. Ampliar estudos em contextos diversos para compreender melhor a variabilidade e aprimorar a comunicação com famílias sobre o processo de fim de vida.
Extubação terminal vs. desmame terminal (E3, E4, E24, E26, E27, E28).	A extubação imediata associa-se a maior frequência de eventos respiratórios e menor tempo para o óbito; o desmame terminal é percebido como menos abrupto e mais aceitável por parte da equipe.	Falta compreensão dos fatores que orientam a escolha entre técnicas, incluindo influências culturais, éticas, institucionais e percepção de familiares.	Ambas as abordagens são eticamente aceitáveis, devendo ser selecionadas conforme quadro clínico, preferências do paciente e protocolos locais.	Desenvolver estudos comparativos de alta qualidade que avaliem desfechos clínicos, experiência dos familiares e percepção dos profissionais.
Sintomas durante a Extubação Paliativa (E20, E22, E23, E31, E44).	O desconforto respiratório é frequente, mas pode ser controlado com uso antecipado de opioides e sedação proporcional, garantindo alívio adequado de sofrimento.	Escassez de métodos padronizados para mensurar sintomas e avaliar a eficácia das intervenções farmacológicas.	O uso ético de sedativos e analgésicos para alívio do sofrimento é justificado, mesmo que possa secundariamente abreviar a vida (princípio do duplo efeito).	Realizar estudos multicêntricos utilizando instrumentos validados, como o RDOS, e promover sua adaptação cultural e linguística para diferentes países.
Vivências, desafios e suporte aos profissionais (E6, E10, E12, E14, E15).	Cresce o reconhecimento da carga emocional, ética e organizacional da EP, com destaque para a importância de equipes integradas, comunicação interdisciplinar e suporte mútuo.	Escassez de estudos quantitativos sobre impacto emocional, fisiológico e ocupacional, além de poucas intervenções avaliadas para redução do sofrimento da equipe.	Apoio psicológico, capacitação técnica e acompanhamento por profissionais experientes reduzem impacto emocional; debriefing e protocolos estruturados aumentam segurança e diminuem conflitos.	Desenvolver estudos longitudinais e testar intervenções estruturadas (simulação, treinamento em CP, suporte psicológico e educacional) voltadas à saúde mental e desempenho das equipes.
Implementação de protocolos (E5, E6, E17, E38).	Protocolos institucionais podem reduzir conflitos, esclarecer condutas e proporcionar maior segurança para pacientes, familiares e profissionais.	Pouca disponibilidade de protocolos validados e baixa adesão institucional, mesmo quando diretriz	Protocolos bem estruturados promovem uniformidade, qualidade do cuidado e conforto durante o processo de EP.	Desenvolver, validar e implementar protocolos multidisciplinares adaptados às realidades culturais e institucionais.
Determinantes da decisão pela Extubação Paliativa (E7, E34, E42).	As decisões são influenciadas por fatores clínicos, contexto institucional, percepção da equipe e preferências do paciente; maior chance de EP em pacientes com menor suporte ventilatório e morte esperada.	Falta de critérios uniformes e pouca compreensão das variações culturais, institucionais e subjetivas que moldam a decisão.	A decisão deve integrar avaliação clínica, preferências do paciente e comunicação estruturada com familiares e equipe, favorecendo decisões compartilhadas.	Investir em estudos multicêntricos que comparem práticas entre instituições e desenvolvam critérios objetivos e ferramentas para apoiar decisões éticas em fim de vida.

## DISCUSSÃO

Ao analisar o conjunto de estudos incluídos, observa-se um aumento expressivo das investigações sobre a EP nos últimos anos. Esse crescimento está diretamente relacionado à ampliação do uso de terapias de suporte vital nas UTI e à necessidade de reavaliar os LSV em situações irreversíveis. Assim, a produção científica recente reflete uma demanda crescente por referenciais teóricos e práticos que orientem decisões clínicas complexas e promovam uma assistência ética, segura e humanizada no fim da vida.

Apesar desse avanço quantitativo, a literatura ainda carece de evidências robustas que subsidiem a padronização da prática da EP. Predominam estudos observacionais, o que limita a generalização dos achados e evidencia a necessidade de investigações com maior rigor metodológico, como ensaios clínicos e estudos de intervenção. Essa limitação é particularmente relevante, considerando que a EP envolve decisões de elevado impacto para pacientes, familiares e equipes multiprofissionais.

Com base na análise das evidências reunidas, a produção científica sobre EP foi organizada em diferentes padrõesanalíticos, que evidenciam tanto os avanços quanto as lacunas na compreensão dessa prática. Entre os padrões identificados, destacam-se as recomendações para a prática clínica na EP. Os estudos convergem para a necessidade de um processo estruturado, que envolva comunicação transparente, manejo rigoroso dos sintomas e suporte contínuo a pacientes, familiares e equipes^(3,10,18,21,26)^. Os achados reforçam o papel central de enfermeiros na coordenação do procedimento, no acolhimento emocional e na promoção de um ambiente de cuidado humanizado^(22,29)^.

Além disso, evidencia-se a importância do planejamento detalhado após a decisão pela EP. Esse planejamento deve incluir reuniões interdisciplinares e com familiares, definição do método (desmame terminal ou extubação imediata), cuidados prévios, estratégias de manejo medicamentoso e cuidados subsequentes^(55)^. O preparo envolve a definição de data e horário, organização da equipe multiprofissional, documentação, adequação do ambiente e orientação ao paciente e à família; já os cuidados posteriores incluem manutenção da sedação, monitoramento de sintomas, eventual transferência de pacientes e apoio às equipes e familiares^(35,55)^.

A condução inadequada da EP pode expor pacientes a complicações importantes, sobretudo o desconforto respiratório, um dos sintomas mais difíceis de manejar e particularmente frequente^(33,43)^. Além do impacto direto sobre o paciente, esse sofrimento repercute de forma significativa sobre familiares e sobre a equipe multiprofissional, ampliando a carga emocional e a complexidade assistencial^(27)^.

Nesse contexto, torna-se evidente que a EP afeta não apenas quem recebe o cuidado, mas também quem o presta: estudos mostram que profissionais de saúde frequentemente vivenciam sofrimento emocional, exaustão e luto persistente ao manejar o processo de morrer, muitas vezes sem suporte institucional adequado^(25,26)^. Diante disso, recomenda-se a adoção de estratégias estruturadas de apoio, como suporte entre pares, grupos reflexivos, recursos psicológicos e espirituais, além de debriefings formais após cada procedimento - a fim de mitigar o desgaste emocional e qualificar o cuidado^(10,25)^.

A literatura também demonstra que a técnica empregada na EP influencia diretamente a expressão de sintomas, o que reforça a necessidade de preparo adequado da equipe. Estudos franceses observaram que a extubação imediata se associou a respiração ofegante, obstrução brônquica e dor, efeitos atenuados quando a sedação profunda foi utilizada^(33)^. Já na Holanda, o uso proporcional de opioides e sedativos manteve baixa a incidência de sofrimento respiratório sem antecipar o óbito, evidenciando que o manejo sintomático adequado é seguro e fundamental para assegurar uma morte digna^(41)^. Esses achados convergem ao indicar que o temor de abreviar a vida não deve restringir o uso de medidas farmacológicas, sob risco de subtratamento e de sofrimento evitável^(10)^.

Estudos brasileiros evidenciam ampla variabilidade no intervalo até a morte após a EP: em um deles^(30)^, a maioria dos pacientes faleceu após 24 horas, com tempo variando de 1 a 19 dias; em outro^(36)^, identificou-se uma média de 2,5 dias entre a extubação e o óbito. Em linha com essa heterogeneidade, evidências internacionais indicam que o tempo até a morte é determinado majoritariamente pela gravidade clínica e por parâmetros fisiológicos pré-extubação - como APACHE II elevado,instabilidade hemodinâmica, uso de vasopressores e necessidade de FiO_2_ alta - e não pelo uso de opioides ou sedativos. Assim, o desfecho imediato reflete principalmente a condição basal do paciente, independentemente da técnica aplicada. Embora a sobrevida varie amplamente, a maioria dos pacientes morre em até 24 horas após a EP, reforçando o papel dos marcadores clínicos prévios como principais preditores do tempo até o óbito^(20,42)^.

Do ponto de vista ético, há consenso de que a sedação proporcional e o uso de analgésicos são moralmente justificáveis quando orientados pelo alívio do sofrimento. Mesmo que tais intervenções possam secundariamente abreviar a vida, sua finalidade primária - controlar dispneia e outros desconfortos - as legitima, desde que aplicadas com intenção clara e documentada^(33)^.

Para avançar na qualidade do cuidado e com o objetivo de auxiliar profissionais de saúde na avaliação da necessidade de titulação de medicamentos para alívio do desconforto durante a EP nos cuidados de fim de vida, Margaret L. Campbell desenvolveu a *Respiratory Distress Observation Scale* (RDOS). Essa escala mensura a presença e a intensidade do desconforto respiratório em pacientes que não conseguem relatar a sensação de dispneia. Cada parâmetro é avaliado com uma pontuação de 0 a 2, sendo os escores somados para obtenção de um valor total que varia de 0, indicando ausência de sofrimento, a 16, representando sofrimento respiratório intenso^(27)^. No entanto, a ausência de validação transcultural dessa escala para o português do Brasil limita sua aplicabilidade na prática nacional.

Quanto aos métodos de descontinuação da ventilação mecânica, não há evidências que indiquem superioridade entre a extubação imediata e o desmame terminal. A extubação imediata tende a ser percebida como um processo mais natural, embora esteja associada a maior risco de desconforto respiratório^(7,55)^. O desmame terminal, por outro lado, é considerado menos abrupto e geralmente mais alinhado às preferências de enfermeiros, técnicos de enfermagem^(37)^ e médicos. Isso é evidenciado em estudo recente que aponta predominância marcante da escolha pelo desmame terminal entre médicos, sendo adotado por 90% dos profissionais, enquanto apenas um optou pela extubação imediata^(56)^.

A decisão entre desmame terminal e extubação imediata deve considerar o nível de suporte ventilatório, o risco de sintomas após a retirada do suporte e as preferências do paciente e da família. O desmame terminal é mais indicado quando o paciente depende de suporte ventilatório moderado a alto, permitindo uma redução progressiva e melhor manejo do conforto. Já a extubação imediata tende a ser apropriada em cenários de baixo suporte e baixa probabilidade de desconforto significativo. Independentemente do método escolhido, a otimização prévia de sedação e analgesia permanece requisito fundamental para garantir conforto e dignidade no processo de extubação paliativa^(10)^.

Os achados desta revisão evidenciam ampla variabilidade na condução da EP, tanto entre países quanto dentro de um mesmo sistema de saúde, influenciada por diferenças institucionais, experiência clínica, valores dos profissionais, preferências familiares e fatores culturais, religiosos e legais. Essa heterogeneidade não decorre apenas das condições clínicas dos pacientes, mas sobretudo do contexto institucional. Pesquisa multicêntrica demonstrou que a probabilidade de EP é mais determinada pelo centro onde o cuidado é prestado do que por parâmetros fisiológicos, com taxas significativamente distintas entre países, o que indica predominância de práticas e culturas locais sobre a individualização da decisão^(19)^.

Em consonância, estudo demonstrou ampla variabilidade global nas práticas de LSV, incluindo a retirada de dispositivos como na EP. As diferenças foram expressivas entre regiões, com maior predominância de abstinência ou retirada de terapias em países do Norte da Europa e Austrália/Nova Zelândia, enquanto América Latina e África apresentaram maior frequência de mortes após ressuscitação cardiopulmonar falhada. Esses padrões refletem influências culturais, institucionais, legais e modelos distintos de tomada de decisão, evidenciando que práticas como a EP são profundamente moldadas pelo contexto^(57)^.

Embora fatores clínicos, como menor suporte ventilatório e expectativa de morte, aumentem a probabilidade de realização da EP, a preponderância do ambiente institucional demonstra que a falta de padronização compromete a previsibilidade e consistência do cuidado, podendo gerar insegurança clínica, conflitos éticos e maior angústia familiar^(19)^.

Nesse cenário, torna-se imprescindível a adoção de protocolos claros e baseados em evidências para reduzir a variabilidade assistencial e promover condutas mais consistentes. Protocolos estruturados favorecem a comunicação multiprofissional, orientam o planejamento do procedimento e garantem que as decisões estejam alinhadas aos valores do paciente e da família, além de minimizarem conflitos e reduzirem o sofrimento emocional da equipe^(10,17,18)^. Dessa forma, a padronização configura um elemento central para qualificar a prática da EP, fortalecendo a segurança, a humanização e a robustez técnica do cuidado de fim de vida.

Diante desse cenário, a EP se configura como um procedimento complexo, permeado por dimensões clínicas, éticas e socioculturais. Essa complexidade exige que os profissionais de UTI recebam capacitação específica, contemplando não apenas aspectos técnicos, mas também habilidades comunicacionais, manejo de conflitos e suporte emocional, assegurando qualidade e segurança em todas as etapas do processo.

A principal limitação desta revisão foi a exclusão de artigos de acesso restrito e a escassez de estudos com alto nível de evidência, especialmente no contexto nacional, o que reflete as dificuldades de realizar pesquisas no fim da vida. Além disso, o recorte em adultos em UTI restringe a generalização dos achados, já que a literatura aponta experiências em contextos pediátricos e domiciliares.

Ainda assim, esta revisão contribui de forma significativa para a prática clínica, o ensino e a pesquisa em cuidados paliativos, saúde e enfermagem. Para a formação acadêmica, aponta a necessidade de estratégias pedagógicas inovadoras que preparem os futuros profissionais para lidar com a complexidade da EP. No campo científico, evidencia prioridades de investigação, como a comparação entre métodos de EP, a adaptação de instrumentos de avaliação, a análise do impacto emocional nos profissionais e o desenvolvimento de protocolos validados que considerem as especificidades culturais e assistenciais de cada contexto.

Para os profissionais de saúde, oferece subsídios para a tomada de decisão ética e segura, orientando o manejo de sintomas, a comunicação com familiares e a integração multiprofissional. Especificamente, com relação aos profissionais de enfermagem, reforça a importância do protagonismo da categoria no manejo dos sintomas, no apoio aos familiares e na comunicação multiprofissional, qualificando o cuidado no fim de vida.

Portanto, os achados desta revisão reforçam a necessidade de fortalecer a base científica que orienta a EP, por meio da produção de evidências robustas e da implementação de estratégias formativas e organizacionais que assegurem qualidade, ética e humanização no cuidado ao fim da vida.

## CONCLUSÃO

A revisão de escopo evidenciou que a EP em UTI constitui uma prática complexa, permeada por desafios clínicos, éticos e organizacionais. Foram identificados padrões consistentes relacionados ao manejo de sintomas, comunicação com familiares, integração multiprofissional e planejamento antecipado do cuidado, evidenciando que a adoção de práticas estruturadas pode qualificar a assistência no fim da vida.

No entanto, observou-se grande variabilidade na prática da EP, ausência de protocolos validados e escassez de estudos robustos, especialmente sobre a comparação entre extubação terminal e desmame terminal. A escolha do método mostrou-se influenciada por fatores clínicos, culturais e institucionais, destacando a necessidade de critérios uniformes e sustentados por evidências.

Embora o manejo farmacológico adequado se mostre eficaz para controlar o desconforto respiratório sem antecipar a morte, o impacto emocional da EP sobre os profissionais permanece pouco explorado, indicando um campo importante para investigação. Assim, recomenda-se o desenvolvimento de ensaios clínicos comparativos, a ampliação de estudos em diferentes contextos assistenciais, a elaboração de protocolos multidisciplinares validados, a adaptação transcultural de instrumentos de avaliação de desconforto e a análise de estratégias de suporte técnico e psicológico para as equipes.

O avanço do conhecimento sobre a EP tem potencial significativo para qualificar os cuidados de fim de vida em terapia intensiva, contribuindo para práticas mais seguras, éticas e alinhadas às necessidades dos pacientes e de suas famílias.

## Data Availability

Todo o conjunto de dados que dá suporte aos resultados deste estudo foi publicado no próprio artigo.

# Translation: Extubación paliativa en unidades de terapia intensiva: revisión de alcance

**DOI:** 10.1590/1980-220X-REEUSP-2025-0409es

**Authors:** Rafaella Guilherme Gonçalves, Lucas Batista Ferreira, Tarcísio Tércio das Neves, Carlos Jordão de Assis Silva, Fabiane Rocha Botarelli, Rejane Maria Paiva de Menezes

**Affiliations:** 1 Universidade Federal do Rio Grande do Norte, Departamento de Enfermagem, Natal, RN, Brasil.

**Keywords:** Cuidados Paliativos, Unidades de Cuidados Intensivos, Grupo de Atención al Paciente, Extubación Traqueal, Enfermería

## Abstract

**Objetivo:**

Mapear el conocimiento existente sobre la extubación paliativa en pacientes adultos ingresados en unidades de terapia intensiva.

**Método:**

Revisión de alcance realizada según las directrices del JBI. Las búsquedas se realizaron entre octubre y diciembre de 2024 en siete bases de datos y cuatro repositorios, además de una búsqueda inversa. La selección fue independiente, la extracción siguió un protocolo estructurado y el informe utilizó PRISMA-ScR. El análisis de los resultados se basó en el marco PAGER (Patterns, Advances, Gaps, Evidence for practice and Research recommendations).

**Resultados:**

La búsqueda identificó 1463 materiales e incluyó 44 estudios. Predominaron las publicaciones del año 2023. Los hallazgos abordaron recomendaciones para la práctica, tiempo hasta la muerte, comparación entre la extubación terminal y el destete terminal, manejo de síntomas, experiencias y desafíos de los profesionales, además de aspectos relacionados con la toma de decisiones y el uso de protocolos en la extubación paliativa.

**Conclusión:**

La extubación paliativa sigue caracterizándose por prácticas heterogéneas y una producción limitada de evidencia de alto nivel. Se necesitan protocolos basados en la evidencia y capacitación multiprofesional para garantizar una atención de calidad, dignidad y alivio del sufrimiento al final de la vida.

## INTRODUCCIÓN

Los pacientes con enfermedades o afecciones que ponen en peligro la vida pueden evolucionar hacia cuadros críticos, caracterizados por inestabilidad o riesgo inminente de fallo orgánico y muerte. En estas situaciones, la asistencia suele prestarse en Unidades de Terapia Intensiva (UTI) o de Cuidados Intermedios (UCI), entornos preparados para ofrecer soporte vital de alta complejidad, con monitorización continua y recursos destinados al mantenimiento de la vida^(1)^. A pesar de los avances tecnológicos, la mortalidad en las UTI brasileñas sigue siendo elevada, oscilando entre el 9,6% y el 58%, influenciada por factores como la gravedad clínica, las comorbilidades, el tiempo de hospitalización, la ventilación mecánica, la sepsis, la insuficiencia renal aguda y la calidad de la atención^(2)^.

La integración temprana de los cuidados paliativos (CP) al tratamiento modificador de la enfermedad es esencial para la atención integral del paciente crítico, contribuyendo a evitar intervenciones inútiles, reducir el tiempo de hospitalización y los costos, aliviar el sufrimiento y preservar la dignidad, sin anticipar el proceso de muerte^(3)^. Cuando se incorporan de forma concomitante, los CP favorecen decisiones éticas y humanizadas, centradas en el control riguroso de los síntomas^(2)^.

Además, la introducción temprana de los CP favorece la definición de los Límites de Soporte Vital (LSV), permitiendo que los profesionales y familiares participen en las decisiones sobre el plan terapéutico y acompañen la progresión de los CP hasta la fase del final de la vida^(4)^. Los LSV consisten en no iniciar, suspender o limitar las intervenciones de soporte vital en situaciones irreversibles, evitando tratamientos fútiles que prolonguen artificialmente el proceso de muerte^(5)^. En el entorno de la UTI, el debate es particularmente relevante, incluyendo decisiones sobre hemodiálisis, fármacos vasoactivos, nutrición artificial y ventilación mecánica invasiva, siendo esta última, por lo general, la medida final que se suspende, ya que su retirada suele determinar la muerte a corto plazo^(6,7)^.

La extubación paliativa (EP) se refiere a la retirada planificada de la ventilación mecánica en pacientes con muerte inminente, y se reconoce como una práctica ética y moralmente justificable, ya que evita la distanasia y permite una muerte cómoda y digna^(8)^. Este procedimiento requiere preparación técnica, comunicación eficaz y actuación multiprofesional cualificada, a fin de garantizar el control de los síntomas y el apoyo adecuado al paciente y a la familia^(9)^.

Sin embargo, la implementación de la EP aún enfrenta desafíos significativos. Entre ellos, se destacan las barreras culturales, religiosas y emocionales, la ausencia de protocolos institucionales claros y la heterogeneidad de conductas entre los equipos^(10)^.

En el contexto brasileño, las dificultades se ven agravadas por las lagunas en la formación académica y profesional sobre el proceso de morir y la muerte, por la insuficiente capacitación en la comunicación de malas noticias y por las limitaciones estructurales para la integración de los CP en la terapia intensiva^(5)^. Así, la PE se configura como una de las conductas de LSV más complejas y sensibles, que exige preparación técnica, ética y emocional de los equipos de salud.

Ante este escenario, es fundamental ampliar el debate y las reflexiones sobre la actuación de los profesionales de la salud en el manejo adecuado de la EP, con especial atención a la enfermería, que desempeña un papel central en la promoción del confort físico, psíquico, social y espiritual de los pacientes al final de la vida. Así, este estudio tiene como objetivo mapear el conocimiento existente sobre la extubación paliativa en pacientes adultos ingresados en unidades de terapia intensiva, sintetizando los hallazgos según el marco PAGER (Patrones, Avances, Lagunas, Evidencia para la práctica y Recomendaciones de investigación).

## MÉTODO

### Diseño del Estudio

Se trata de una revisión de alcance, que buscó explorar, mapear, sintetizar la evidencia científica e identificar las lagunas de conocimiento sobre la EP en pacientes adultos ingresados en la UTI. El método siguió las directrices del JBI para revisiones de alcance^(11)^ y la redacción cumplió con las recomendaciones del PRISMA-ScR^(12)^. El protocolo de esta revisión se registró en el Open Science Framework (DOI: 10.17605/OSF.IO/JYMNZ).

### Recolección de Datos

Inicialmente, se realizó una búsqueda preliminar en las bases de datos MEDLINE y Scopus con el objetivo de identificar estudios relacionados con el tema, así como seleccionar palabras clave y términos de índice utilizados en estas publicaciones. Para la elaboración de la pregunta orientadora, se adoptó el acrónimo PCC, descrito en el Cuadro 1.

**Cuadro 1:** Estrategia PCC (Población, Concepto y Contexto) – Natal, RN, Brasil, 2025.

Sigla	Término	Descripción
P	Población	Pacientes adultos en cuidados de fin de vida
C	Concepto	Extubación paliativa
C	Contexto	Unidades de terapia intensiva

A partir de esta estrategia, se formuló la siguiente pregunta de investigación: ¿Qué conocimientos existen en la literatura sobre la extubación paliativa de pacientes adultos ingresados en unidades de cuidados intensivos?.

La revisión bibliográfica se llevó a cabo entre octubre y diciembre de 2024 a través del portal de revistas de la Coordinación de Perfeccionamiento del Personal de Nivel Superior (CAPES), vía Comunidad Académica Federada (CAFe), contemplando las bases SCOPUS (Elsevier), MEDLINE/PubMed (Biblioteca Nacional de Medicina), Web of Science, CINAHL (Índice acumulativo de literatura de *enfermería y salud afín*), *Google Scholar*, LILACS (Literatura Latinoamericana y del Caribe en Ciencias de la Salud) y BDENF (Base de Datos de Enfermería). De manera complementaria, se realizó una búsqueda inversa en las referencias de los artículos incluidos, con el fin de identificar estudios adicionales pertinentes. Para ampliar el alcance, también se consultaron fuentes de literatura gris, incluyendo tesis y disertaciones disponibles en repositorios en línea: Catálogo de Tesis y Disertaciones de CAPES, BASE (*Bielefeld Academic Search Engine*), *Cybertesis* y *Open Access Theses & Dissertations* (OATD).

Los descriptores seleccionados para realizar la búsqueda fueron los términos de los Descriptores en Ciencias de la Salud (DeCS) y los correspondientes en los *Medical Subject Headings* (MESH): Cuidados Paliativos/Palliative Care, Extubación/Airway Extubation y Unidades de Terapia Intensiva/Intensive Care Units, además de las palabras clave: Terminal Care, End-of-life care, Compassionate Extubation, Terminal weaning y Terminal extubation y la palabra clave: Extubación Paliativa.

Las estrategias de búsqueda se elaboraron y adaptaron de acuerdo con las especificidades sintácticas y operativas de cada base de datos, respetando los respectivos campos de indexación, el uso de operadores booleanos y los recursos propios de recuperación de información. El Cuadro 2 presenta las estrategias de búsqueda adoptadas en cada base de datos.

**Cuadro 2:** Estrategia de búsqueda utilizada para cada base de datos – Natal, RN, Brasil, 2025.

Base	Estrategia
SCOPUS	(“Palliative Care” OR “Terminal Care” OR “End-of-life Care”) AND (“Airway Extubation” OR “Compassionate Extubation” OR “Terminal Extubation” OR “Terminal Weaning”) AND (“Intensive Care Units”)
MEDLINE/PubMed	(Palliative Care OR Terminal Care OR End-of-life Care) AND (Airway Extubation OR Compassionate Extubation OR Terminal Extubation OR Terminal Weaning) AND (Intensive Care Units)
Web of Science	ALL=(Palliative Care OR Terminal Care OR End-of-life Care) AND ALL=(Airway Extubation OR Compassionate Extubation OR Terminal Extubation OR Terminal Weaning) AND ALL=(Intensive Care Units)
CINAHL	(Palliative Care OR Terminal Care OR End-of-life Care) AND (Airway Extubation OR Compassionate Extubation OR Terminal Extubation OR Terminal Weaning) AND (Intensive Care Units)
Google Scholar	(“Palliative Care” OR “Terminal Care” OR “End-of-life Care”) AND (“Airway Extubation” OR “Compassionate Extubation” OR “Terminal Extubation” OR “Terminal Weaning”) AND (“Intensive Care Units”)
LILACS	Palliative Care AND Airway Extubation
BDENF	Palliative Care AND Airway Extubation
**Literatura Gris**	**Estrategia**
Catálogo de teses e dissertações da CAPES	Extubação Paliativa
BASE	Palliative Care AND Airway Extubation
Cybertesis	Palliative Care AND Airway Extubation
OATD	Palliative Care AND Airway Extubation AND Intensive Care Units

### Criterios de Selección

Los criterios de selección se establecieron en función de la pregunta orientadora, teniendo en cuenta la estrategia PCC. Se consideraron estudios con las siguientes características: a) en cuanto a la población: estudios realizados con pacientes en cuidados paliativos, mayores de 18 años; b) en cuanto al concepto: estudios que evaluaron o describieron la realización de la EP; c) en cuanto al contexto: estudios que contemplaran la EP realizada en las UTI. Además, se incluyeron estudios de investigación primaria con diseños cuantitativos, cualitativos y de métodos mixtos, estudios de revisión y de opinión clínica, disponibles en su totalidad, sin restricción de idioma y sin límite temporal. Se excluyeron resúmenes y cartas al editor.

### Extracción y ANálisis de Datos

La selección de los estudios fue realizada por dos revisores, de forma independiente y ciega, en dos fases: selección de títulos/resúmenes y lectura íntegra de los textos potencialmente elegibles. Se eliminaron los duplicados y, en caso de desacuerdo, se consultó a un tercer revisor. Además, se realizó una búsqueda inversa en las referencias de los estudios incluidos. El proceso de identificación, selección e inclusión se detalla en un diagrama de flujo de acuerdo con la guía PRISMA-ScR^(12)^.

Para la extracción de datos, se elaboró un instrumento de recolección que contenía las variables de interés: autores, año de publicación, país, objetivos, método, población y tamaño de la muestra, resultados y principales hallazgos relacionados con el tema de la revisión. La información extraída se organizó en una hoja de cálculo y se presentó mediante estadísticas descriptivas. Además, se elaboró un cuadro resumen para facilitar la visualización y síntesis de los hallazgos.

En cuanto al análisis, se utilizó la metodología PAGER (Patrones, Avances, Lagunas, Evidencia para la práctica y Recomendaciones de investigación), que amplía la calidad y el rigor de las revisiones de alcance mediante un análisis estructurado de los hallazgos. Este enfoque contempla: (1) Patrones: principales temas emergentes; (2) Avances: nuevos descubrimientos, conocimientos teóricos o metodológicos; (3) Lagunas: áreas que requieren investigaciones adicionales; (4) Evidencia para la práctica: hallazgos relevantes para la aplicación práctica; y (5) Recomendaciones de investigación: orientaciones para futuros estudios^(13)^.

### Aspectos Éticos

La investigación se llevó a cabo de acuerdo con los principios éticos y metodológicos aplicables, respetando las buenas prácticas científicas y los derechos de autor de las obras consultadas. Al tratarse de una revisión basada en datos secundarios de acceso público, sin participación directa de seres humanos, no fue necesario someterla al comité de ética en investigación.

## RESULTADOS

La búsqueda realizada entre octubre y diciembre de 2024 identificó 1463 registros, de los cuales 1389 procedían de bases de datos y 74 de literatura gris. Tras la selección de títulos y resúmenes, se excluyeron 1389 estudios por no cumplir los criterios de elegibilidad, quedando 74 para su lectura en texto completo. En la siguiente etapa, se eliminaron 26 duplicados (23 de las bases de datos y 3 de la literatura gris), lo que dio un total de 48 estudios elegibles. De estos, ocho fueron excluidos por no presentar el escenario de realización o por alejarse del tema propuesto. Tras la lectura íntegra, se incluyeron 40 estudios, a los que se sumaron cuatro identificados mediante búsqueda manual en las referencias. Así, la muestra final comprendió 44 estudios, tal y como se ilustra en el diagrama de flujo PRISMA-ScR (Figura 1).

**Figura 1:** Diagrama de flujo PRISMA-ScR de selección de publicaciones – Natal, RN, Brasil, 2025.

Los 44 estudios incluidos en esta revisión de alcance consisten en publicaciones de los últimos 25 años, comprendidos entre 1999 y 2024, con predominio de siete estudios (15,9%) publicados en 2023. Además, 2024 ya se presenta como un año prometedor para la investigación sobre el tema, ya que se han identificado seis publicaciones (13,6%) realizadas en ese período. Esta información se presenta en el Cuadro 3.

**Cuadro 3:** Caracterización de los estudios en función del autor, año, país de origen, objetivo, diseño del estudio, población y principales resultados – Natal, RN, Brasil, 2025.

Id	Autores/Año/País	Tipo de estudio, población y muestra	Principales resultados
E1	Jennerich, 2024^(3)^. EE. UU.	Revisión bibliográfica, 83 estudios	El control adecuado de los síntomas y la preparación del equipo son esenciales para minimizar el sufrimiento durante y después de la EP.
E2	Schaden et al., 2024^(14)^. Austria	Revisión bibliográfica, no describe	La transición temprana a los cuidados paliativos favorece la dignidad al final de la vida; la EP requiere una preparación rigurosa.
E3	Kumar et al., 2024^(15)^. India	Revisión bibliográfica, 15 estudios	La PE y el destete terminal son opciones éticas cuando se ajustan a los intereses del paciente terminal.
E4	Curtisi et al., 2024^(16)^. EE. UU	Informe de caso, 2 pacientes	El destete gradual asociado con un tratamiento adecuado de los síntomas proporcionó comodidad, mientras que la extubación brusca con medicación insuficiente provocó sufrimiento.
E5	Marica, 2024^(17)^. EE. UU.	Estudio de intervención	Una guía multidisciplinar basada en la evidencia para la EP aumenta la seguridad profesional y la calidad de la atención.
E6	Bryan et al., 2024^(18)^. EE. UU.	Observacional prospectivo, con enfoque cualitativo, 312 profesionales sanitarios	La comunicación y la integración multiprofesional, combinadas con el uso de protocolos, son fundamentales para una EP segura y humanizada, siendo la relación entre el equipo y la familia un elemento central en el proceso de toma de decisiones al final de la vida.
Y 7	Waldauf et al., 2023^(19)^. Canadá, Chequia y Países Bajos	Estudio observacional prospectivo, 616 pacientes	La decisión de realizar una EP se vio influida por el contexto institucional y se asoció con un menor soporte ventilatorio y una menor supervivencia.
E8	Zheng et al., 2023^(20)^. Taiwán	Estudio observacional retrospectivo, 140 pacientes	El tiempo hasta la muerte tras la EP fue variable, siendo más corto en pacientes con mayor gravedad clínica, lo que ayuda a planificar los cuidados paliativos.
E9	Mazzu et al., 2023^(21)^. EE. UU.	Revisión bibliográfica, 73 estudios	La comunicación estructurada y el apoyo del equipo son prácticas clave; las pruebas sólidas siguen siendo limitadas.
E10	Ortega-Chen et al., 2023^(10)^. EE. UU.	Revisión bibliográfica, no describe	La EP es un proceso complejo y variable que requiere habilidades de comunicación paliativa, una planificación cuidadosa, la evaluación y el manejo de los síntomas y la realización de sesiones informativas.
E11	Roberts y Dahlin, 2023^(22)^. EE. UU	Revisión narrativa, no describe	Las enfermeras desempeñan un papel fundamental y pueden liderar prácticas basadas en la evidencia en la EP.
E12	Bastos et al., 2023^(23)^. Brasil	Descriptiva, con un enfoque cualitativo, profesionales de la salud	Los profesionales reconocen los beneficios de la EP, pero informan de inseguridad técnica y necesidad de formación.
E13	Antonio y Antonio, 2023^(24)^. Brasil	Estudio observacional prospectivo, 18 pacientes	La implementación de un protocolo local organizó la práctica y mostró variabilidad en el tiempo hasta la muerte.
E14	Kaur et al., 2022^(25)^. EE. UU.	Transversal, 20 profesionales sanitarios	La EP se asoció con un malestar emocional significativo entre los profesionales sanitarios, especialmente entre las mujeres jóvenes y aquellos insatisfechos con el proceso institucional.
E15	Orr et al., 2022^(26)^ EE. UU.	Descriptivo, con un enfoque cualitativo, 20 profesionales sanitarios: médicos, enfermeros y fisioterapeutas.	Las experiencias profesionales influyen en la realización de la EP; la formación y el apoyo reducen los conflictos.
E16	Cooney-Newton y Hare, 2022^(27)^. EE. UU.	Revisión bibliográfica, no describe	La Escala de Observación de Dificultad Respiratoria es una herramienta objetiva que ayuda a ajustar la medicación para aliviar el malestar durante los cuidados paliativos y la EP.
E17	Correa-Pérez, 2022^(28)^. España	Revisión bibliográfica, no describe	Se necesitan protocolos institucionales para la sedación, la analgesia y el apoyo a las familias.
E18	Dias, 2021^(29)^. Brasil	Revisión integradora, 20 estudios	La enfermería es fundamental en la toma de decisiones, la realización de la EP y el mantenimiento del confort.
E19	Lacerda et al., 2020^(30)^. Brasil	Observacional retrospectivo, 31 pacientes	La EP no aumentó la mortalidad hospitalaria y mostró una amplia variabilidad en el tiempo hasta la muerte.
E20	Fehnel et al., 2020^(31)^. EE. UU.	Observacional retrospectivo, 822 pacientes	La administración temprana de opioides reduce el riesgo de taquipnea grave, especialmente en pacientes con mayor gravedad clínica.
E21	Efstathiou et al., 2020^(32)^. Canadá	Revisión sistemática de métodos mixtos, 25 estudios	Existe una gran variabilidad en las prácticas, siendo el destete terminal el enfoque más común, mientras que la extubación terminal es más frecuente en América del Norte y se asocia con una mayor intensidad de los síntomas respiratorios.
E22	Robert et al., 2020^(33)^. Francia	Estudio observacional prospectivo, 450 pacientes	La sedación profunda y el uso de vasopresores se asociaron con menos molestias tras la retirada de la ventilación mecánica.
E23	Yeh et al., 2019^(34)^. EE. UU	Estudio observacional retrospectivo, 38 pacientes	La alta frecuencia de angustia después de la EP indica la necesidad de una sedación más eficaz.
E24	Janssens, 2017^(35)^. Francia	Observacional prospectivo, 458 pacientes y familiares.	La extubación inmediata causó más dificultad respiratoria que la retirada gradual terminal.
E25	Rebelatto y Moritz, 2017^(36)^. Brasil.	Estudio observacional retrospectivo, 23 pacientes	La supervivencia tras la EP se relacionó con la gravedad de la enfermedad, no con el procedimiento en sí.
E26	Robert et al., 2017^(7)^. Francia	Estudio observacional prospectivo, 458 pacientes sometidos a EP, sus familias y profesionales	La extubación inmediata no alteró el bienestar psicológico de los familiares, pero se asoció con una mayor dificultad respiratoria en los pacientes en comparación con el destete terminal.
E27	Thellier et al., 2017^(37)^. Francia	Estudio observacional retrospectivo, 68 pacientes	La extubación inmediata se asoció con un tiempo más corto hasta la muerte, mientras que el destete terminal fue mejor aceptado por el personal de enfermería.
E28	Cottereau et al., 2016^(38)^. Francia	Estudio transversal, 451 profesionales sanitarios (enfermeros y médicos)	La preferencia por el destete terminal se asoció con la falta de conocimiento o la percepción desfavorable de la EP.
E29	Delaney y Downar, 2016^(39)^. Canadá	Revisión integradora, 99 estudios	Gran variabilidad en la retirada del soporte vital debido a la escasez de pruebas sólidas.
E30	Graeff et al., 2016^(40)^. Países Bajos	Revisión bibliográfica, no describe	Existe una escasez de protocolos estructurados para los cuidados al final de la vida en la UCI.
E31	Epker et al., 2015^(41)^. Países Bajos.	Estudio observacional prospectivo, 241 pacientes	El uso progresivo de opioides y sedantes se asoció con una muerte rápida y pocas molestias.
E32	Long et al., 2015^(42)^. EE. UU.	Estudio observacional retrospectivo, 330 pacientes	La mayoría de los pacientes fallecieron en el plazo de una hora o hasta 24 horas después de retirar la ventilación mecánica, y el tiempo más corto hasta la muerte se asoció con la presencia de diabetes y valores más altos de presión positiva al final de la espiración.
E33	Campbell et al., 2015^(43)^. EE. UU.	Casi experimental, 14 pacientes	El algoritmo dirigido por enfermeras mejoró la comodidad respiratoria durante la EP.
E34	Huynh et al., 2013^(44)^. EE. UU.	Observacional retrospectivo, 322 pacientes	La EP fue más frecuente en pacientes de edad avanzada y con antecedentes de muerte prevista, y fue menos común en los servicios quirúrgicos; el menor tiempo hasta la muerte se asoció con el uso de vasopresores y fracciones elevadas de oxígeno inspirado.
E35	Epker et al., 2011^(45)^. Países Bajos	Estudio observacional retrospectivo, 80 pacientes	La retirada combinada de la ventilación y los fármacos vasoactivos redujo el tiempo hasta la muerte.
E36	Mazer et al., 2011^(46)^. EE. UU.	Estudio observacional prospectivo, 74 pacientes	Las dosis paliativas más altas de opioides se asociaron con un tiempo más prolongado hasta la muerte.
E37	Cooke et al., 2010^(47)^. EE. UU.	Estudio observacional retrospectivo, 1505 pacientes	Las variables demográficas y las medidas de la gravedad de la enfermedad subyacente explican en parte el tiempo transcurrido hasta la muerte después de la EP.
E38	Kompanje et al., 2008^(48)^. EE. UU.	Revisión bibliográfica, no describe	El protocolo por fases favoreció el control de los síntomas y una muerte tranquila.
E39	Fartoukh et al., 2005^(49)^. Francia	Observacional retrospectivo, 5 pacientes	La extubación terminal demostró ser viable y transparente en pacientes con daño neurológico irreversible, con muerte en los tres días siguientes al procedimiento.
E40	Rocker et al., 2004^(50)^. Canadá.	Estudio observacional prospectivo, 206 pacientes	La mayoría de los pacientes fallecieron tras la extubación, con el uso predominante de morfina, midazolam y lorazepam, y los familiares informaron de que se sintieron cómodos independientemente de la técnica utilizada o del tiempo transcurrido hasta la muerte.
E41	Chan et al., 2004^(51)^. EE. UU.	Estudio observacional retrospectivo, 75 pacientes	Los narcóticos y las benzodiazepinas no aceleraron la muerte tras la EP.
E42	Cook et al., 2003^(52)^. Canadá, EE. UU., Suecia, Australia.	Estudio observacional prospectivo, 851 pacientes	La retirada del soporte vital se vio influida principalmente por el criterio médico clínico y la percepción de una baja probabilidad de supervivencia, lo que refuerza la importancia de tener en cuenta las preferencias del paciente al final de su vida.
E43	Kirchhoff et al., 2003^(53)^. EE. UU.	Descriptivo, con un enfoque cuantitativo, 31 enfermeras	Las enfermeras desempeñan un papel fundamental en la preparación de las familias para la retirada del respirador.
E44	Mayer y Kossoff, 1999^(54)^. EE. UU.	Observacional retrospectivo, 74 pacientes	Se realizó la extubación terminal en el 43% de los pacientes, con supervivencia variable, signos respiratorios frecuentes y uso predominante de opioides para aliviar las molestias, y el proceso fue bien aceptado por los familiares.

En cuanto a la ubicación de los estudios, la mayoría se desarrollaron en Estados Unidos (EE. UU.), con 21 estudios (47,7%), seguidos de Francia, con seis (13,6%), y Brasil, con cinco (11,4%). Canadá y los Países Bajos presentaron tres estudios cada uno (6,8%), mientras que Austria, India, Taiwán y España contribuyeron con un estudio cada uno (2,3%). Cabe destacar que los porcentajes relativos al país en el que se realizaron los estudios no son mutuamente excluyentes, ya que algunos estudios se llevaron a cabo en más de un país. Además, dos estudios (4,5%) se realizaron en diferentes países y, en general, cuatro (9,1%) fueron investigaciones multicéntricas.

En cuanto al diseño de los estudios, predominaron los estudios observacionales, con un total de 25 publicaciones (56,8%), de las cuales 13 (29,5%) eran de cohortes retrospectivas, 10 (22,7%) de cohortes prospectivas y dos estudios transversales (4,5%). A continuación, destacan los estudios de revisión, que corresponden a 13 publicaciones (29,5%). También se identificaron tres estudios descriptivos (6,8%) y, por último, un informe de caso, un estudio cuasi experimental y un estudio de intervención, cada uno de los cuales representa el 2,3% de la muestra. Las principales características metodológicas de los artículos analizados se presentan en el Cuadro 3.

La evidencia resumida en el Cuadro 4 se analizó utilizando la metodología PAGER, lo que permitió una comprensión estructurada de los avances y las lagunas en la producción científica sobre la EP en la UCI. Se identificaron siete patrones principales: recomendaciones para la práctica de la extubación paliativa (14 estudios), variabilidad en el tiempo hasta la muerte tras la extubación paliativa (11 estudios), comparación entre la extubación terminal y el destete terminal (6 estudios), síntomas durante la extubación paliativa (5 estudios), experiencias, retos y apoyo a los profesionales (5 estudios), implementación de protocolos (4 estudios) y determinantes de la decisión de extubación paliativa (3 estudios). Estos hallazgos ponen de relieve la complejidad del proceso de extubación paliativa y la diversidad de dimensiones clínicas, éticas y organizativas abordadas en la literatura.

**Cuadro 4:** Síntesis del conocimiento guiada por el marco PAGER – Natal, RN, Brasil, 2025

Normas	Avances	Lagunas	Evidencia para la práctica	Recomendaciones para la investigación
Recomendaciones para la práctica en la extubación paliativa (E1, E2, E9, E10, E11, E16, E18, E21, E29, E30, E33, E38, E39, E43).	Reconocimiento cada vez mayor de la necesidad de una planificación estructurada, un tratamiento adecuado de los síntomas, una comunicación clara con los familiares y un trabajo interprofesional coordinado.	Gran variabilidad entre instituciones y falta de directrices clínicas sólidas y estandarizadas para el manejo de la EP en la UCI.	Las prácticas basadas en la evidencia, como la evaluación sistemática de los síntomas, los protocolos de comunicación y el apoyo del equipo, reducen los conflictos, aumentan la seguridad y mejoran la experiencia del paciente y la familia.	Realizar estudios multicéntricos sólidos para apoyar el desarrollo de guías clínicas estandarizadas aplicables a diferentes contextos institucionales.
Variabilidad en el tiempo hasta la muerte tras la extubación paliativa (E8, E13, E25, E32, E34, E35, E36, E37, E40, E41, E44).	La mayoría de los pacientes fallecen en las 24 horas siguientes a la EP, a pesar de la variabilidad individual. Los factores predictivos de una muerte más rápida incluyen un APACHE II alto, vasopresores, FiO2 alta, inestabilidad hemodinámica y compromiso respiratorio. Los opioides y los sedantes proporcionales no aceleran la muerte.	La heterogeneidad metodológica, las muestras limitadas y las diferencias clínicas dificultan la generalización de los resultados. No hay suficientes estudios sobre los factores culturales y organizativos que pueden influir en la supervivencia tras la EP.	El tiempo hasta la muerte depende de la gravedad clínica pre extubación, y no de la analgesia o la sedación. Los predictores de muerte temprana ayudan a planificar los cuidados, preparar al equipo y comunicarse de forma realista con los familiares.	Desarrollar y validar modelos pronósticos multicéntricos para estimar el tiempo hasta la muerte tras la EP. Ampliar los estudios en diferentes contextos para comprender mejor la variabilidad y mejorar la comunicación con las familias sobre el proceso del final de la vida.
Extubación terminal frente a retirada terminal (E3, E4, E24, E26, E27, E28).	La extubación inmediata se asocia con una mayor frecuencia de eventos respiratorios y un tiempo más corto hasta la muerte; el destete terminal se percibe como menos abrupto y más aceptable por el equipo.	Existe una falta de comprensión de los factores que guían la elección entre las técnicas, incluidas las influencias culturales, éticas e institucionales y las percepciones de la familia.	Ambos enfoques son éticamente aceptables y deben seleccionarse en función del estado clínico, las preferencias del paciente y los protocolos locales.	Desarrollar estudios comparativos de alta calidad que evalúen los resultados clínicos, la experiencia de los familiares y las percepciones de los profesionales.
Síntomas durante la extubación paliativa (E20, E22, E23, E31, E44).	La dificultad respiratoria es frecuente, pero puede controlarse con el uso temprano de opioides y sedación proporcional, lo que garantiza un alivio adecuado del sufrimiento.	Escasez de métodos estandarizados para medir los síntomas y evaluar la eficacia de las intervenciones farmacológicas.	El uso ético de sedantes y analgésicos para aliviar el sufrimiento está justificado, aunque pueda acortar secundariamente la vida (principio del doble efecto).	Realizar estudios multicéntricos utilizando instrumentos validados, como el RDOS, y promover su adaptación cultural y lingüística para diferentes países.
Experiencias, retos y apoyo a los profesionales (E6, E10, E12, E14, E15).	Cada vez se reconoce más la carga emocional, ética y organizativa de la EP, haciendo hincapié en la importancia de los equipos integrados, la comunicación interdisciplinaria y el apoyo mutuo.	Existen pocos estudios cuantitativos sobre el impacto emocional, fisiológico y ocupacional, así como pocas intervenciones evaluadas para reducir el sufrimiento del personal.	El apoyo psicológico, la formación técnica y la supervisión por parte de profesionales experimentados reducen el impacto emocional; las sesiones informativas y los protocolos estructurados aumentan la seguridad y reducen los conflictos.	Desarrollar estudios longitudinales y probar intervenciones estructuradas (simulación, formación en PC, apoyo psicológico y educativo) dirigidas a la salud mental y al rendimiento del equipo.
Implementación de protocolos (E5, E6, E17, E38).	Los protocolos institucionales pueden reducir los conflictos, aclarar la conducta y proporcionar mayor seguridad a los pacientes, las familias y los profesionales.	Escasa disponibilidad de protocolos validados y baja adherencia institucional, incluso cuando existen directrices.	Los protocolos bien estructurados promueven la uniformidad, la calidad de la atención y la comodidad durante el proceso de PE.	Desarrollar, validar e implementar protocolos multidisciplinarios adaptados a las realidades culturales e institucionales.
Determinantes de la decisión de extubación paliativa (E7, E34, E42).	Las decisiones están influenciadas por factores clínicos, el contexto institucional, la percepción del personal y las preferencias del paciente; mayor probabilidad de extubación e en pacientes con menos apoyo ventilatorio y muerte prevista.	Falta de criterios uniformes y escasa comprensión de las variaciones culturales, institucionales y subjetivas que configuran la decisión.	La decisión debe integrar la evaluación clínica, las preferencias del paciente y la comunicación estructurada con los familiares y el personal, favoreciendo las decisiones compartidas.	Invertir en estudios multicéntricos que comparen las prácticas entre instituciones y desarrollen criterios y herramientas objetivos para apoyar las decisiones éticas al final de la vida.

## DISCUSIÓN

Al analizar el conjunto de estudios incluidos, se observa un aumento significativo de las investigaciones sobre la EP en los últimos años. Este crecimiento está directamente relacionado con la ampliación del uso de terapias de soporte vital en las UTI y con la necesidad de reevaluar los LSV en situaciones irreversibles.

Así, la producción científica reciente refleja una creciente demanda de referencias teóricas y prácticas que orienten las decisiones clínicas complejas y promuevan una atención ética, segura y humanizada al final de la vida.

A pesar de este avance cuantitativo, la literatura aún carece de evidencias sólidas que respalden la estandarización de la práctica de la EP. Predominan los estudios observacionales, lo que limita la generalización de los hallazgos y evidencia la necesidad de investigaciones con mayor rigor metodológico, como ensayos clínicos y estudios de intervención. Esta limitación es particularmente relevante, considerando que la AP implica decisiones de gran impacto para los pacientes, sus familiares y los equipos multidisciplinarios.

Basándose en el análisis de las evidencias reunidas, la producción científica sobre la AP se organizó en diferentes patrones analíticos, que evidencian tanto los avances como las lagunas en la comprensión de esta práctica. Entre los patrones identificados, destacan las recomendaciones para la práctica clínica en la AP. Los estudios coinciden en la necesidad de un proceso estructurado, que implique una comunicación transparente, un manejo riguroso de los síntomas y un apoyo continuo a los pacientes, familiares y equipos^(3,10,18,21,26)^. Los hallazgos refuerzan el papel central de los enfermeros en la coordinación del procedimiento, el apoyo emocional y la promoción de un entorno de cuidados humanizados^(22,29)^.

Además, se evidencia la importancia de una planificación detallada tras la decisión de la PE. Esta planificación debe incluir reuniones interdisciplinarias y con los familiares, la definición del método (destete terminal o extubación inmediata), los cuidados previos, las estrategias de manejo farmacológico y los cuidados posteriores^(55)^. La preparación implica definir la fecha y la hora, organizar al equipo multidisciplinario, documentar, adaptar el entorno y orientar al paciente y a la familia; por su parte, los cuidados posteriores incluyen mantener la sedación, monitorear los síntomas, la posible transferencia de pacientes y el apoyo a los equipos y familiares^(35,55)^.

La conducción inadecuada de la EP puede exponer a los pacientes a complicaciones importantes, sobre todo a la dificultad respiratoria, uno de los síntomas más difíciles de manejar y particularmente frecuente^(33,43)^. Además del impacto directo sobre el paciente, este sufrimiento repercute de manera significativa en los familiares y en el equipo multidisciplinario, lo que aumenta la carga emocional y la complejidad de la atención^(27)^.

En este contexto, resulta evidente que los cuidados paliativos no solo afectan a quienes reciben la atención, sino también a quienes la prestan: los estudios muestran que los profesionales de la salud a menudo experimentan sufrimiento emocional, agotamiento y duelo persistente al manejar el proceso de la muerte, a menudo sin el apoyo institucional adecuado^(25,26)^. Ante esto, se recomienda la adopción de estrategias de apoyo estructuradas, como el apoyo entre pares, grupos de reflexión, recursos psicológicos y espirituales, además de sesiones informativas formales después de cada procedimiento, con el fin de mitigar el desgaste emocional y mejorar la calidad de la atención^(10,25)^.

La literatura también demuestra que la técnica empleada en la EP influye directamente en la expresión de los síntomas, lo que refuerza la necesidad de una preparación adecuada del equipo. Estudios franceses observaron que la extubación inmediata se asoció con respiración jadeante, obstrucción bronquial y dolor, efectos que se atenuaron cuando se utilizó sedación profunda^(33)^. En los Países Bajos, el uso proporcional de opioides y sedantes mantuvo baja la incidencia de sufrimiento respiratorio sin anticipar la muerte, lo que demuestra que el manejo sintomático adecuado es seguro y fundamental para garantizar una muerte digna^(41)^. Estos hallazgos coinciden al indicar que el temor a acortar la vida no debe restringir el uso de medidas farmacológicas, bajo riesgo de subtratamiento y sufrimiento evitable^(10)^.

Estudios brasileños evidencian una amplia variabilidad en el intervalo hasta la muerte después de la EP: en uno de ellos^(30)^, la mayoría de los pacientes fallecieron después de 24 horas, con un tiempo que varió de 1 a 19 días; en otro^(36)^, se identificó un promedio de 2,5 días entre la extubación y la muerte.

En consonancia con esta heterogeneidad, las evidencias internacionales indican que el tiempo hasta la muerte está determinado principalmente por la gravedad clínica y los parámetros fisiológicos previos a la extubación, como APACHE II elevado, inestabilidad hemodinámica, uso de vasopresores y necesidad de FiO_2_ alta, y no por el uso de opioides o sedantes. Por lo tanto, el resultado inmediato refleja principalmente el estado basal del paciente, independientemente de la técnica aplicada. Aunque la supervivencia varía ampliamente, la mayoría de los pacientes fallecen en las 24 horas siguientes a la EP, lo que refuerza el papel de los marcadores clínicos previos como principales predictores del tiempo hasta la muerte^(20,42)^.

Desde el punto de vista ético, existe consenso en que la sedación proporcional y el uso de analgésicos son moralmente justificables cuando se orientan al alivio del sufrimiento. Aunque estas intervenciones pueden acortar secundariamente la vida, su finalidad principal—controlar la disnea y otras molestias—las legitima, siempre que se apliquen con una intención clara y documentada^(33)^.

Para mejorar la calidad de la atención y con el objetivo de ayudar a los profesionales de la salud a evaluar la necesidad de titular los medicamentos para aliviar las molestias durante la EP en los cuidados al final de la vida, Margaret L. Campbell desarrolló la Escala de Observación de la Dificultad Respiratoria (RDOS). Esta escala mide la presencia y la intensidad de las molestias respiratorias en pacientes que no pueden informar de la sensación de disnea. Cada parámetro se evalúa con una puntuación de 0 a 2, y las puntuaciones se suman para obtener un valor total que varía de 0, lo que indica ausencia de sufrimiento, a 16, lo que representa un sufrimiento respiratorio intenso^(27)^. Sin embargo, la falta de validación transcultural de esta escala para el portugués de Brasil limita su aplicabilidad en la práctica nacional.

En cuanto a los métodos de desconexión de la ventilación mecánica, no hay pruebas que indiquen la superioridad de la extubación inmediata sobre el destete terminal. La extubación inmediata tiende a percibirse como un proceso más natural, aunque se asocia a un mayor riesgo de malestar respiratorio^(7,55)^. El destete terminal, por otro lado, se considera menos abrupto y generalmente más acorde con las preferencias de enfermeros, técnicos de enfermería^(37)^ y médicos. Esto se evidencia en un estudio reciente que señala una marcada predominancia de la elección del destete terminal entre los médicos, adoptado por el 90% de los profesionales, mientras que solo uno optó por la extubación inmediata^(56)^.

La decisión entre el destete terminal y la extubación inmediata debe tener en cuenta el nivel de soporte ventilatorio, el riesgo de síntomas tras la retirada del soporte y las preferencias del paciente y la familia. El destete terminal es más adecuado cuando el paciente depende de un soporte ventilatorio moderado a alto, lo que permite una reducción progresiva y un mejor manejo del confort. Por otro lado, la extubación inmediata tiende a ser adecuada en situaciones de bajo soporte y baja probabilidad de malestar significativo. Independientemente del método elegido, la optimización previa de la sedación y la analgesia sigue siendo un requisito fundamental para garantizar el confort y la dignidad en el proceso de extubación paliativa^(10)^.

Los hallazgos de esta revisión evidencian una amplia variabilidad en la realización de la PE, tanto entre países como dentro de un mismo sistema de salud, influenciada por diferencias institucionales, experiencia clínica, valores de los profesionales, preferencias familiares y factores culturales, religiosos y legales. Esta heterogeneidad no se debe solo a las condiciones clínicas de los pacientes, sino sobre todo al contexto institucional. Una investigación multicéntrica demostró que la probabilidad de la PE está más determinada por el centro donde se presta la atención que por los parámetros fisiológicos, con tasas significativamente diferentes entre países, lo que indica el predominio de las prácticas y culturas locales sobre la individualización de la decisión^(19)^.

En consonancia, un estudio demostró una amplia variabilidad global en las prácticas de LSV, incluida la retirada de dispositivos como en la PE. Las diferencias fueron significativas entre regiones, con una mayor predominancia de abstinencia o retirada de terapias en países del norte de Europa y Australia/Nueva Zelanda, mientras que América Latina y África presentaron una mayor frecuencia de muertes tras una reanimación cardiopulmonar fallida. Estos patrones reflejan influencias culturales, institucionales, legales y modelos distintos de toma de decisiones, lo que evidencia que prácticas como la EP están profundamente moldeadas por el contexto^(57)^.

Aunque factores clínicos, como un menor soporte ventilatorio y la expectativa de muerte, aumentan la probabilidad de realizar la PE, la preponderancia del entorno institucional demuestra que la falta de estandarización compromete la previsibilidad y la consistencia de la atención, lo que puede generar inseguridad clínica, conflictos éticos y mayor angustia familiar^(19)^.

En este escenario, es imprescindible adoptar protocolos claros y basados en la evidencia para reducir la variabilidad asistencial y promover conductas más consistentes. Los protocolos estructurados favorecen la comunicación multiprofesional, orientan la planificación del procedimiento y garantizan que las decisiones estén alineadas con los valores del paciente y la familia, además de minimizar los conflictos y reducir el sufrimiento emocional del equipo^(10,17,18)^. De este modo, la estandarización constituye un elemento central para calificar la práctica de la EP, fortaleciendo la seguridad, la humanización y la solidez técnica de los cuidados al final de la vida.

Ante este escenario, la EP se configura como un procedimiento complejo, impregnado de dimensiones clínicas, éticas y socioculturales. Esta complejidad exige que los profesionales de la UTI reciban una formación específica, que contemple no solo aspectos técnicos, sino también habilidades comunicativas, manejo de conflictos y apoyo emocional, garantizando la calidad y la seguridad en todas las etapas del proceso.

La principal limitación de esta revisión fue la exclusión de artículos de acceso restringido y la escasez de estudios con un alto nivel de evidencia, especialmente en el contexto nacional, lo que refleja las dificultades de realizar investigaciones sobre el final de la vida. Además, el enfoque en adultos en la UTI restringe la generalización de los hallazgos, ya que la literatura señala experiencias en contextos pediátricos y domiciliarios.

Aun así, esta revisión contribuye de manera significativa a la práctica clínica, la enseñanza y la investigación en cuidados paliativos, salud y enfermería. En cuanto a la formación académica, señala la necesidad de estrategias pedagógicas innovadoras que preparen a los futuros profesionales para lidiar con la complejidad de la EP. En el campo científico, evidencia prioridades de investigación, como la comparación entre métodos de EP, la adaptación de instrumentos de evaluación, el análisis del impacto emocional en los profesionales y el desarrollo de protocolos validados que consideren las especificidades culturales y asistenciales de cada contexto.

Para los profesionales de la salud, ofrece subsidios para la toma de decisiones éticas y seguras, orientando el manejo de los síntomas, la comunicación con los familiares y la integración multiprofesional. Específicamente, en relación con los profesionales de enfermería, refuerza la importancia del protagonismo de la categoría en el manejo de los síntomas, el apoyo a los familiares y la comunicación multiprofesional, calificando el cuidado al final de la vida.

Por lo tanto, los hallazgos de esta revisión refuerzan la necesidad de fortalecer la base científica que orienta la EP, mediante la producción de evidencias sólidas y la implementación de estrategias formativas y organizativas que garanticen la calidad, la ética y la humanización en los cuidados al final de la vida.

## CONCLUSIÓN

La revisión del alcance evidenció que la EP en la UTI constituye una práctica compleja, impregnada de retos clínicos, éticos y organizativos. Se identificaron patrones consistentes relacionados con el manejo de los síntomas, la comunicación con los familiares, la integración multiprofesional y la planificación anticipada de los cuidados, lo que evidencia que la adopción de prácticas estructuradas puede mejorar la asistencia al final de la vida.

Sin embargo, se observó una gran variabilidad en la práctica de la PE, la ausencia de protocolos validados y la escasez de estudios sólidos, especialmente sobre la comparación entre la extubación terminal y el destete terminal. La elección del método se vio influida por factores clínicos, culturales e institucionales, lo que pone de relieve la necesidad de criterios uniformes y respaldados por pruebas.

Aunque el manejo farmacológico adecuado se muestra eficaz para controlar el malestar respiratorio sin anticipar la muerte, el impacto emocional de la PE sobre los profesionales sigue siendo poco explorado, lo que indica un campo importante para la investigación. Por lo tanto, se recomienda el desarrollo de ensayos clínicos comparativos, la ampliación de estudios en diferentes contextos asistenciales, la elaboración de protocolos multidisciplinarios validados, la adaptación transcultural de instrumentos de evaluación del malestar y el análisis de estrategias de apoyo técnico y psicológico para los equipos.

El avance del conocimiento sobre la EP tiene un potencial significativo para mejorar la calidad de los cuidados al final de la vida en terapia intensiva, contribuyendo a prácticas más seguras, éticas y alineadas con las necesidades de los pacientes y sus familias.

## Data Availability

Todo el conjunto de datos que apoya los resultados de este estudio fue publicado en el propio artículo.
